# Parkinson's‐Linked *LRRK2* and *GBA1* Mutations Modulate the Peripheral Immune Response to *Pseudomonas aeruginosa*

**DOI:** 10.1002/mds.70123

**Published:** 2025-11-19

**Authors:** Julian R. Mark, Hannah A. Staley, Ann M. Titus, Julian Agin‐Liebes, Alicia Garrido, Laura Hughes, Nicolas Dzamko, Rebecca L. Wallings, Malú Gámez Tansey

**Affiliations:** ^1^ Department of Neuroscience, University of Florida College of Medicine Gainesville FL USA; ^2^ Center for Translational Research in Neurodegenerative Disease, University of Florida College of Medicine Gainesville FL USA; ^3^ Trinity College of Arts and Sciences, Duke University Durham NC USA; ^4^ Department of Neurology Columbia University Irving Medical Center New York NY USA; ^5^ Parkinson's Disease and Movement Disorders Unit Institut Clínic de Neurociències Hospital Clinic de Barcelona Barcelona Spain; ^6^ Medicine and Health University of Sydney Sydney NSW Australia; ^7^ Department of Neurology Indiana University School of Medicine Indianapolis IN USA; ^8^ Stark Neuroscience Research Institute Indianapolis IN USA

**Keywords:** Parkinson's Disease, inflammation, LRRK2, GBA1, glucocerebrosidase

## Abstract

**Background:**

Peripheral disease mechanisms such as immune dysregulation may contribute to Parkinson's disease (PD). To investigate interactions between common PD mutations and immune responses to environmental pathogens, we studied responses to *Pseudomonas aeruginosa* (*P. aeruginosa*) in peripheral blood mononuclear cells (PBMCs) from PD patients with *leucine‐rich repeat kinase 2* ( *LRRK2*) mutations, *GBA1* mutations, and idiopathic disease (iPD) relative to neurologically healthy controls (NHC).

**Objectives:**

The goal was to test the hypothesis that *LRRK2* and *GBA* modify the human peripheral immune response to bacteria, specifically *P. aeruginosa*, based on prior animal studies involving *Lrrk2* mutations and microbial pathogens.

**Methods:**

PBMCs from *LRRK2* ‐PD, *GBA* ‐PD, and iPD patients plus age‐ and sex‐matched controls were treated ex vivo with live *P. aeruginosa* and pharmacological agents that block LRRK2 kinase activity (MLi‐2) or enhance glucocerebrosidase (GCase) activation (NCGC00188758) to measure enzymatic activities and cytokine release.

**Results:**

*GBA* ‐PD PBMCs exhibited increased *P. aeruginosa*‐dependent secretion of specific inflammatory cytokines including interleukin‐1β. Antigen presentation was increased in *LRRK2* ‐PD nonclassical monocytes treated with the GCase activator. Levels of pRab10, a proxy for LRRK2 kinase activity, were increased in *GBA* ‐PD classical monocytes relative to NHC and iPD. GCase activator treatment increased pRab10 expression in *LRRK2* ‐PD intermediate monocytes. *GBA* ‐PD and individual treatments with MLi‐2 or GCase activator were associated with reduced *P. aeruginosa*‐dependent LRRK2 protein levels in PBMC subsets.

**Conclusions:**

This work demonstrates that PD‐linked mutations in *LRRK2* and *GBA1* converge on peripheral blood immune cell dysregulation, as evinced by the ability of LRRK2 inhibitors and GCase activators to modulate the ex vivo immune response to bacterial exposure. © 2025 The Author(s). *Movement Disorders* published by Wiley Periodicals LLC on behalf of International Parkinson and Movement Disorder Society.

Parkinson's disease (PD) is a multi‐system neurodegenerative disease for which there are no effective disease‐modifying therapies. Both central and peripheral immune dysregulation have been implicated in PD,^1^ and this has led to growing interest in peripheral immune dysfunction as a potential contributor to PD pathogenesis. For example, PD is associated with dysregulated expression of tumor necrosis factor (TNF) and interferon‐γ (IFNγ),^2^ and individuals with PD show reduced numbers of circulating regulatory T cells.^3^, ^4^ Furthermore, monocytes from PD patients display a hyperinflammatory response to ex vivo stimulation with lipopolysaccharide (LPS), which positively correlates with Hoehn and Yahr score of motor impairment.^5^ Together, these findings point toward widespread changes across both the innate and adaptive peripheral immune system in PD. Consistent with this idea, biomarkers of systemic inflammation like the neutrophil‐to‐lymphocyte ratio are significantly correlated with lower levels of dopamine transporter in the striatum of PD patients.^6^ Therefore, peripheral immune dysregulation may be a critical driver for neuronal degeneration in PD and investigating this mechanism has potential to reveal novel methods for earlier diagnosis, delaying, or slowing disease progression.

One of the most commonly mutated genes in familial PD, *leucine‐rich repeat kinase 2* (*LRRK2*), has been extensively linked to endolysosomal pathway regulation and immune cell function.^7^, ^8^, ^9^ The predominant PD‐associated mutation in *LRRK2* is the glycine‐to‐serine substitution at amino acid 2019 (*G2019S*), which is a gain‐of‐kinase activity mutation.^8^ *Lrrk2‐G2019S* mutation leads to increased interleukin (IL)‐1β production in mice in response to *Salmonella typhimurium* infection and improves survival.^10^, ^11^ Interestingly, *Lrrk2* knockout (KO) in mice renders them more susceptible to intestinal infection with *Listeria monocytogenes*,^12^ but it enhances the immune response to *Mycobacterium tuberculosis* and reduces bacterial burden.^13^ These results indicate that the role of *LRRK2* in pathogen control and immune function may vary depending on the identity of the pathogen.

Another significant genetic risk factor for PD is mutations in *GBA1*, the gene that encodes glucocerebrosidase (GCase).^14^ GCase is a lysosomal hydrolase that catalyzes the cleavage of glucosylceramide into ceramide and glucose,^15^ and similarly to *LRRK2*, mutations in *GBA1* have been implicated in immune function.^16^ For example, *GBA‐N370S* increases resistance to *Mycobacterium tuberculosis* in zebrafish through enhancing the microbicidal activity of macrophage lysosomes.^17^ In addition, *GBA1* deletion in *Drosophila* causes immune hyperactivation and microbial dysbiosis through autophagic defects.^18^ In humans, both *GBA1* and *LRRK2* mutations have been associated with changes in the levels of circulating cytokine levels in the blood,^19^, ^20^, ^21^ but it remains to be determined whether *LRRK2* or *GBA1* mutations modulate the immune response to pathogens in peripheral blood cells.

Much of the previous literature on peripheral inflammation in PD and *LRRK2/GBA1* mutations in humans has focused on circulating cytokines in the plasma or serum at a single time point when an individual blood sample is drawn. Circulating cytokines are subject to significant variability associated with circadian rhythm, sleep, diet, recent environmental exposures, and more.^22^, ^23^, ^24^ In contrast, ex vivo stimulation‐based assays may mitigate these factors by measuring immune “traits” defined by stimulus‐evoked activation and resolution responses, and they can be reproducibly elicited in a controlled ex vivo experiment regardless of exogenous factors. Moreover, epidemiological studies have associated exposure to environmental factors, including pathogens, with a heightened long‐term risk of developing PD,^25^ suggesting that experiments aimed at identifying immune dysfunction traits may reveal differences between cases and controls. In addition, using a range of LPS‐, cytokine‐, and bead‐based stimulations may help reveal shifts in sensitivity threshold to specific immune challenges that can help ex vivo evaluation of immune effector functions in subsets of peripheral blood immune cell populations.

Therefore, we sought to investigate whether the response to a bacterial pathogen was dysregulated in peripheral blood immune cells from PD patients carrying pathogenic *LRRK2* or *GBA1* mutations relative to idiopathic PD (iPD) or neurologically healthy controls (NHCs). To this end, we developed a protocol for ex vivo stimulation of plated human peripheral blood mononuclear cells (PBMCs) using live bacteria. We selected *Pseudomonas aeruginosa* (*P. aeruginosa*) as the model microbe for this study because of its clinical relevance as a common cause of nosocomial infections^26^ and its known ability to induce neuroinflammation and blood–brain barrier dysfunction, which are processes that are heavily implicated in PD pathogenesis.^27^ Specifically relevant to PD, *P. aeruginosa* produces biosurfactants that facilitate α‐synuclein–mediated membrane permeabilization and neurotoxicity,^28^ and others have demonstrated that PINK1/Parkin are involved in regulating the immune response to gram‐negative bacteria like *P. aeruginosa*.^29^ These findings motivated us to optimize a protocol for stimulation with *P. aeruginosa*, and we applied this protocol to study immune responses in PBMCs from individuals with *LRRK2*‐PD, *GBA‐*PD, iPD, or matched NHCs. In addition, we tested whether pharmacologic inhibition of LRRK2 kinase activity with MLi‐2 or enhancement of GCase activity with NCGC00188758 (NCG) would modulate the response to *P. aeruginosa*. We observed enhanced secretion of IL‐1β from *GBA*‐PD PBMCs after *P. aeruginosa* treatment, and enhancement of GCase activity with NCG elicited increased IL‐2 secretion from *GBA*‐PD cells relative to other groups. Major histocompatibility complex class II (MHC‐II) expression in response to *P. aeruginosa* was increased in *LRRK2*‐PD nonclassical monocytes when treated with GCase activator and MLi‐2 and NCG modulated antigen presentation with cell‐type specific effects. Notably, both MLi‐2 and NCG caused treatment‐specific effects on the expression of LRRK2 and phosphorylated Rab10 (pRab10), a bona fide LRRK2 kinase substrate.^30^ Our results reveal that *GBA*‐PD and *LRRK2*‐PD patients display distinct signatures of peripheral immune cell activation relative to NHCs and iPD patients, and these pathogenic mutations may converge on common pathways to contribute to immune dysregulation in PD.

## Materials and Methods

### Human Subjects

This study was reviewed and approved by the University of Florida institutional review board (IRB202001775) and institutional review boards at Columbia and Barcelona. Participants provided written informed consent to participate after being informed of the risks and benefits associated with their participation. Blood was initially collected from healthy control individuals to establish and optimize assay parameters, followed by recruitment of subjects with PD and neurologically healthy controls through sites at Columbia University Irving Medical Center, NYC and the Hospital Clinic de Barcelona. Recruitment was conducted from 2021 to 2023. A licensed neurologist performed a clinical evaluation to obtain Unified Parkinson's Disease Rating Scale scores, and this information is displayed in Table S1. Whole blood was collected from participants, and then PBMCs were isolated and cryopreserved as described below. Genotyping was completed on site to determine the presence of *LRRK2* and *GBA1* mutations, and controls were also genotyped to confirm the absence of *LRRK2* or *GBA1* mutations. All *LRRK2*‐PD individuals were confirmed to have the *G2019S* mutation. The frequency of different *GBA1* mutations (eg, N370S, V394L, E326K, and L444P) in the *GBA‐*PD cohort is described in Table S1. During recruitment, a questionnaire was used to assess for sex, age, age at disease onset, and disease duration. NHC individuals between 30 and 80 years old were recruited. Controls had no known familial PD mutations and were without known neurological illness, chronic or recent infections, or autoimmune comorbidities.

### PBMC Isolation and Cryopreservation

Cell isolation was performed using previously established methods^34^ using BD Vacutainer CPT Cell Preparation Tube with Sodium Citrate (BD Biosciences, Franklin Lakes, New Jersey, 362761). Approximately six CPT tubes, each containing 8 mL of blood, were collected from each participant. CPT tubes were inverted 8 to 10 times and centrifuged at 1500 × *g* for 20 minutes at room temperature. The PBMC enriched layer was transferred to a new 50 mL conical tube and magnetic‐activated cell sorting (MACS) buffer (phosphate‐buffered saline [PBS], 0.5% bovine serum albumin [BSA], 20 mM EDTA, pH 7.2) was added to a final volume of 50 mL, followed by centrifugation at 1800 × *g* for 10 minutes at room temperature. Following removal of the supernatant, PBMCs were resuspended in 10 mL MACS buffer and counted on a hemocytometer using Trypan blue (1:20 dilution) exclusion to ascertain viability.

Next, to cryopreserve the samples, PBMCs were centrifuged for 5 minutes 1800 × *g* at room temperature. Supernatant was aspirated and cell pellets were gently resuspended in cryopreservation media (54% Roswell Park Memorial Institute [RPMI] 1640, 36% fetal bovine serum [FBS], 10% dimethyl sulfoxide [DMSO]) at a final concentration of 1 × 10^7^ cells/mL in cryovials (Simport, T311‐2). Cryovials were placed in a room‐temperature Mr. Frosty freezing container with isopropanol as per manufacturer's instructions and stored at −80°C overnight. After overnight storage at −80°C, cryovials were removed from freezing containers the next day and placed in liquid nitrogen for long‐term storage.

### Cryorecovery of Isolated PBMCs

From cryorecovery onward, researchers were blinded to the disease and mutation status of samples. For cryorecovery, cryovials of PBMCs were retrieved from liquid nitrogen, rapidly thawed in a water bath at 37°C, and rapidly added to 25 mL of 37°C filter sterilized complete culture media (RPMI 1640 media, 10% low endotoxin heat‐inactivated FBS, 1 mM penicillin–streptomycin). PBMCs were pelleted *via* centrifugation at 300 × *g* for 10 minutes at room temperature. Pellets were gently resuspended in 10 mL of 37°C MACS buffer (PBS, 0.5% BSA, 20 mM EDTA, pH 7.2), then viability and cell count were obtained with a hemocytometer using Trypan blue (1:20 dilution) exclusion.

### Optimization of NCG Treatment with of NHCs PBMCs

PBMCs from 10 NHCs were cryorecovered as previously described and plated at a final concentration of 1 × 10^6^ cells/mL in 500 uL in 24‐well plates and allowed to rest for 3 hours. Cells were then treated for 18 hours with either a vehicle control, 50 uM conduritol B‐epoxide (CBE), 100 uM NCG, or 50 uM CBE + 100 uM NCG. Cells were then collected and centrifuged in Eppendorf's at 300 × *g* for 10 minutes at 4° and resuspended in 100 uL of plating media containing 0.375 mM 5‐Pentafluorobenzoylamino Fluorescein Di‐Beta‐D‐Glucopyranoside (PFB‐FDGlu) for 1 hour at 37°. Cells were centrifuged at 300 × *g* for 5 minutes at 4°C, supernatant removed, pellets resuspended in 200 uL PBS and centrifuged at 300 × *g* for 5 minutes at 4°C. Cells were resuspended in live/dead aqua and incubated at room temp for 20 minutes. Cells were washed ×2 in 200 uL PBS and resuspended in 100 uL fluorescence‐activated cell sorting (FACS) buffer and GCase mean fluorescence intensity (MFI) analyzed in live cells on the Macs Quant v10 flow cytometer (Miltenyi Biotec, Bergisch Gladbach, Germany, 130‐096‐343). GBA index was calculated by using the MFI of cleaved GCase substrate PFB‐FDglu in vehicle or NCG‐treated conditions and subtracting the background MFI observed in the conduritol B‐epoxide‐treated conditions for each sample.

### Treatment of PBMCs with *P. Aeruginosa* and MLi‐2/NCG

PBMCs were cryorecovered as previously described, plated to a final concentration of 1 × 10^6^ per mL in 1 mL complete culture media in 24‐well plates, and allowed to rest for 2 hour at 37°C in 5% CO_2_, 95% relative humidity. After resting, cells were treated with live *P. aeruginosa* (cultured from American Type Culture Collection, Manassas, Virginia, reference no. R4605210, Strain 9027, Lot 451 997) at MOI of 10 bacteria per PBMC. Simultaneously with *P. aeruginosa*, PBMCs were also incubated with one of the following drug conditions: equivalent volume of DMSO (for vehicle condition), 100 nM of MLi‐2 in DMSO, or 100 μM of NCG in DMSO. Cells were cultured for 24 hour at 37°C, 5% CO_2_, 95% relative humidity, followed by flow cytometric analysis. Cultured media was retained for analysis of cytokine secretion.

### Fixed Cell Flow Cytometry and Staining Panel for pRab10 and LRRK2

After the 24‐hour stimulation, cells were harvested and centrifuged at 300 × *g* for 5 minutes at 4°C. Supernatant was collected to quantify cytokine secretion (described below). Cell pellets were gently resuspended in 200 μL of cold PBS and transferred to a v‐bottom 96‐well plate (Sigma, Albuquerque, New Mexico, CLS3896‐48EA). Samples were centrifuged at 300 × *g* for 5 minutes at 4°C. Cells were resuspended in 50 μL of Live/Dead Fixable Aqua stain (diluted 1:2000 in PBS, Invitrogen, Waltham, Massachusetts, L34957) with diluted antibodies and incubated in the dark at 4°C for 30 minutes. Antibody cocktail contained anti‐CD3 (PE conjugate, 1:10 dilution, BD Biosciences, 555340), anti‐CD14 (APC‐Vio770 conjugate, 1:50 dilution, Miltenyi Biotec, 130–113‐144), anti‐CD16 (V450 conjugate, 1:20 dilution, BD Biosciences, BDB560474), and anti‐HLA‐DR (PE‐Vio770 conjugate, 1:100 dilution, Miltenyi Biotec, 130–113‐403).

Next, cells were centrifuged at 300 × *g* for 5 minutes at 4°C and washed ×2 in PBS. Cells were re‐suspended and fixed in 100 μL of 1% paraformaldehyde (PFA) at 4°C in the dark for 30 minutes. Cells were washed 2× with PBS, then resuspended in 100 μL of permeabilization buffer (eBiosciences, San Diego, California, 00–8333‐56) and incubated on ice for 15 minutes. Anti‐pT73 Rab10 antibody (Abcam, Cambridge, UK, ab241060) was added to each well at 0.55 μg per well and incubated at room temperature and protected from light for 30 minutes. Cells were centrifuged at 300 × *g* for 5 minutes at 4°C washed in PBS ×2. Cells were resuspended in 100 μL of PBS containing 1% normal goat/donkey serum, 2% BSA and 1:1000 AF488 donkey anti‐rabbit secondary (Thermo Fisher, A‐21206) and incubated at room temperature and protected from light for 30 minutes. Cells were centrifuged at 300 × *g* for 5 minutes at 4°C washed in PBS ×2. Cells were resuspended in 100 μL of PBS containing 1% normal goat/donkey serum, 2% BSA 1:100 anti‐LRRK2 AF700 antibody (Novus Biologicals, Centennial, Colorado, NB300‐268AF700) and incubated at 4°C covered for 20 minutes. Cells were centrifuged at 300 × *g* for 5 minutes at 4°C and then washed in FACS buffer (PBS, 0.5 mM EDTA, 0.1% sodium azide) ×3. Cells were analyzed via flow cytometry on a MACSQuant Analyzer 10 (Miltenyi Biotec). Data were analyzed using FlowJo version 10.10.0 software.

### Cytokine Quantification

V‐PLEX custom Human Biomarkers kit (Meso Scale Discovery [MSD], Rockville, Maryland, K151ARH‐2) was used to quantify cytokines in conditioned media from cultured T cells and monocytes. Media was diluted 1:4 with MSD kit diluent and incubated in duplicate at room temperature in the provided MSD plate with capture antibodies for 2 hours as per manufacturer's instructions. Plates were then washed ×3 with PBS with 0.05% Tween‐20 and detection antibodies conjugated with electrochemiluminescent labels were added and incubated at room temperature for another 2 hours. After 3× washes with PBS containing 0.05% Tween‐20, MSD read buffer was added and the plates were loaded into the QuickPlex MSD instrument for quantification.

### Statistics

Data and statistical analyses were performed using GraphPad Prism 10. For assessing differences between groups, data were analyzed by either one‐way or two‐way analysis of variance (ANOVA), or by *t* test. In instances when data did not fit parametric assumptions, Kruskal–Wallis non‐parametric ANOVA was used. Post hoc tests following ANOVAs were conducted using Tukey's method for correcting for multiple comparisons. For assessing relationships between read‐outs, data were analyzed by Pearson's *r*. In instances when data did not fit parametric assumptions, Spearman's rank was used to assess relationships between variables. Two‐tailed levels of significance were used and *P* < 0.05 was considered statistically significant. Outliers were tested for using iterative Grubbs test (α = 0.01), and outliers were removed before final analysis. Graphs are depicted by means ± standard error of the mean. Experiments were performed using the largest sample size possible (determined by availability of patient samples and recruitment limitations) while maintaining age‐ and sex‐matched groups. Group sizes are listed in all figure legends.

## Results

### Optimized Treatment Parameters for *P. Aeruginosa* Stimulation Provide Robust Immune Response

Previous work by our research group showed that the *Lrrk2‐G2019S* mutation is protective against infection with *P. aeruginosa*,^31^ with *Lrrk2‐G2019S* mice showing improved survival relative to B6 and mice overexpressing wild‐type *Lrrk2*. This motivated us to develop a protocol for ex vivo stimulation of human PBMCs using live *P. aeruginosa* to interrogate the peripheral blood immune cell response. To optimize stimulation parameters, PBMCs from NHCs were cryorecovered (as described under Materials and Methods) and plated to a concentration of 1 million cells/mL in complete growth medium and allowed to rest for 2 hours. Next, live *P. aeruginosa* were added to achieve varying multiplicities of infection (MOIs) ranging from 0.1 to 10. A treatment duration of 24 hours was chosen based on previously reported treatment paradigms.^32^, ^33^ The workflow for these experiments is shown in Figure S1. We determined that an MOI of 10 bacterial cells per PBMC was effective for producing a robust immune response in terms of increasing MHC‐II expression in PBMCs and inflammatory cytokine secretion while not significantly reducing PBMC viability (Fig. S2). Based on these data, we proceeded with an MOI of 10 for downstream and more streamlined application of *P. aeruginosa* to PD patient samples.

### *GBA* ‐PD Peripheral Immune Cells Exhibit Increased Cytokine Secretion in Response to *P. Aeruginosa* Relative to iPD and *LRRK2* ‐PD

**FIG. 1:**
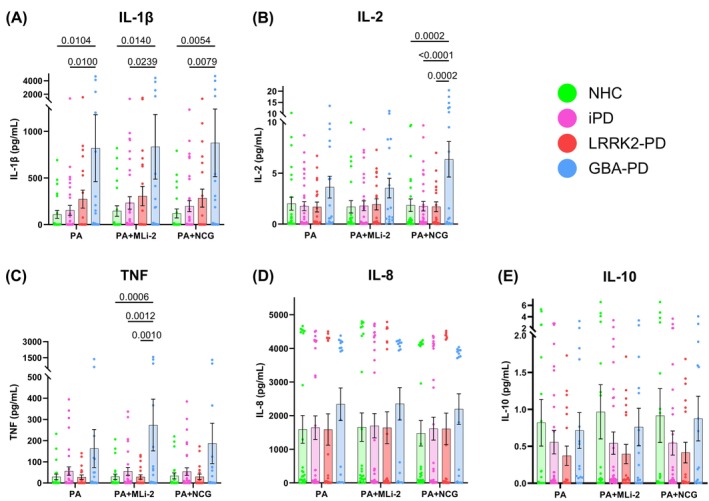
*GBA*‐Parkinson's disease (PD) peripheral immune cells exhibit greater stimulation‐evoked secretion of proinflammatory cytokines. Bar graphs overlaid with scatter plots showing secretion of inflammatory cytokines after *Pseudomonas aeruginosa* treatment from peripheral blood mononuclear cells from neurologically healthy controls (NHCs), idiopathic PD (iPD) patients, *LRRK2‐*PD patients, and *GBA*‐PD patients. (A) Secretion of interleukin (IL)‐1β (effect of interaction *P* > 0.9999, treatment *P* = 0.9246, cohort *P* < 0.0001). (B) IL‐2 (effect of interaction *P* = 0.3396, treatment *P* = 0.3138, cohort *P* < 0.0001). (C) Secretion of tumor necrosis factor (effect of interaction *P* = 0.8677, treatment *P* = 0.6192, cohort *P* < 0.0001). (D) Secretion of IL‐8 (effect of interaction *P* > 0.9999, treatment *P* = 0.9295, cohort *P* = 0.1874). (E) Secretion of IL‐10 (effect of interaction *P* = 0.9997, treatment *P* = 0.9156, cohort *P* = 0.0570). Bars represent mean ± standard error of the mean. NHCs, n = 24; iPD, n = 33; *LRRK2‐*PD, n = 21; *GBA‐*PD, n = 17. Each symbol represents the measurement from a single individual. The results in A–E were analyzed using two‐way analysis of variance with Tukey's corrections for multiple comparisons. Only comparisons within treatment and comparisons within cohorts across treatment are shown. Statistically significant differences defined by *P* < 0.05 are shown. [Color figure can be viewed at wileyonlinelibrary.com]

We next sought to determine if baseline levels of cytokine secretion from PBMCs were affected by *LRRK2* or *GBA1* mutations. To accomplish this, we collected PBMCs from a pilot cohort of NHC, iPD, *LRRK2‐*PD, and *GBA‐*PD individuals. Patients were enrolled across two sites: Columbia University Irving Medical Center in New York City and the Hospital Clinic de Barcelona in Spain. As expected from previous work,^34^ we observed that baseline cytokine secretion in unstimulated PBMCs was not significantly different across groups (Fig. S3). We also observed significant effects of *P. aeruginosa* stimulation on antigen presentation, pRab10 expression, and LRRK2 levels in monocyte subtypes (Fig. S4). This pilot study informed our decision to include pathogen stimulation in all conditions for the subsequent primary experiments. A primary cohort of participants was then recruited from the same sites as the pilot cohort. Whole blood samples were collected from iPD patients (n = 33), *LRRK2*‐PD patients (n = 21), *GBA*‐PD patients (n = 17), and age‐ and sex‐matched NHCs (n = 24). The *LRRK2‐*PD cohort uniformly carried the *G2019S* mutation, whereas the *GBA1‐*PD cohort was a mixed cohort that included *N370S*, *V394L*, *E326K*, or *L444P* mutations in *GBA1* (frequency of each mutation is listed in Table S1). Participants were genotyped to confirm the specific *LRRK2* and *GBA1* mutations that were present, as well as to ensure iPD patients and NHCs were not carriers of these mutations. Full inclusion and exclusion criteria are described in the Methods section (demographic information of the cohorts and mutation frequencies for each cohort are shown in Table S1). From whole blood, PBMCs were isolated and cryopreserved as described in Methods and based on previously published techniques.^34^ In addition, MLi‐2, an inhibitor of LRRK2 kinase activity, and NCG, which enhances GCase activity, were included as treatment conditions in addition to *P. aeruginosa* to determine the role of LRRK2 kinase or GCase activity, respectively, on the immune response to pathogens. Our group has previously demonstrated the ability of MLi‐2 to inhibit LRRK2 kinase activity in vitro,^34^ and for this study we confirmed that NCG was effective in enhancing GCase activity in NHC PBMCs (Fig. S5). We observed that in response to *P. aeruginosa*, IL‐1β secretion was significantly increased in *GBA*‐PD relative to NHC (cohort effect *P* < 0.0001) (Fig. 1A). *GBA*‐PD secretion of IL‐1β remained increased relative to NHC and iPD in MLi‐2 and NCG treatment conditions (Fig. 1A). Interestingly, although IL‐2 secretion was not significantly different between patient cohorts in *P. aeruginosa* alone and MLi‐2 treatment conditions, *GBA*‐PD PBMCs displayed significantly increased secretion of IL‐2 relative to all other patient cohorts in the NCG‐treated condition (cohort effect *P* < 0.0001) (Fig. 1B). TNF secretion was significantly increased in *GBA*‐PD relative to all other cohorts in MLi‐2‐treated conditions (cohort effect *P* < 0.0001) (Fig. 1C). No significant differences between treatment or patient groups were observed in IL‐8 or IL‐10 secretion (Fig. 1D,E). Collectively, these data support a pattern of upregulated secretion of specific proinflammatory cytokines in *GBA*‐PD in response to *P. aeruginosa* exposure.

### PBMC Subtype Frequencies Are Not Significantly Different across *LRRK2‐* PD and *GBA* ‐PD

The capacity to secrete inflammatory cytokines varies with immune cell subtype,^35^ therefore, we sought to investigate if the observed differences in *P. aeruginosa‐*evoked cytokine secretion were secondary to underlying differences in peripheral immune cell frequencies in the blood across cohorts. To assess this, we processed the stimulated PBMCs for flow cytometric analysis using antibody‐fluorophore conjugates for cell‐surface markers. Classical monocytes (CD14^+^/CD16^−^) are the most abundant monocyte subset and play a key role in phagocytosis, inflammation, and migration to sites of infection or injury, where they can differentiate into macrophages or dendritic cells.^36^ Intermediate monocytes (CD14^+^/CD16^+^) produce high levels of inflammatory cytokines, highly express MHC‐II, and are involved potentiating inflammatory responses.^37^, ^38^ Nonclassical monocytes (CD14^−^/CD16^+^) patrol the endothelium and are involved in tissue surveillance and repair, contributing to the resolution of inflammation and vascular homeostasis.^39^ We observed no differences across cohorts in the frequencies of CD3^+^ T cells, classical monocytes (CD14^+^/CD16^−^), intermediate monocytes (CD14^+^/CD16^+^), or nonclassical monocytes (CD14^−^/CD16^+^) (Fig. S6A–D). The frequency of cells that were lacking expression of both T cell and monocyte markers (CD3^−^/CD14^−^/CD16^−^) was lower in *GBA‐*PD relative to iPD (Fig. S6E). These results indicate that *LRRK2*‐PD and *GBA*‐PD diagnoses do not affect PBMC population makeup and differences in cytokine secretion across patient cohorts are not a result of differences in PBMC subtype frequency.

### NCG Treatment Can Modulate Monocyte Antigen Presentation in Response to Pathogen Stimulation in Nonclassical Monocytes

**FIG. 2:**
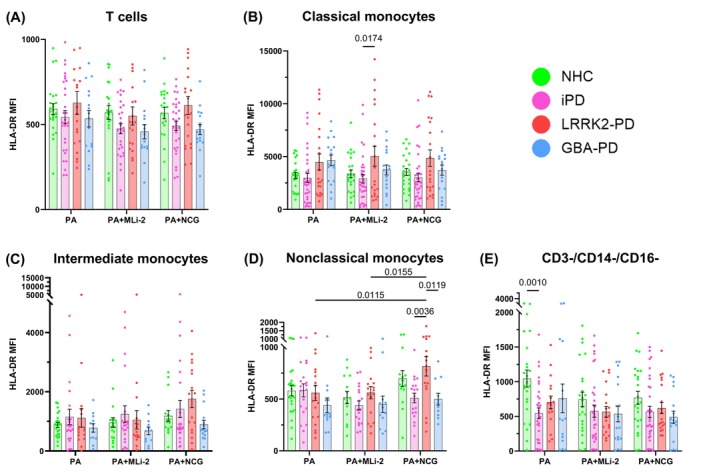
Antigen presentation in response to *Pseudomonas aeruginosa* (*P. aeruginosa*) is modulated by glucocerebrosidase (GCase) activity. Bar graphs overlaid with scatter plots showing expression of major histocompatibility complex class II (MHC‐II) human leukocyte antigen (HLA)‐DR in peripheral blood mononuclear cells from neurologically healthy controls (NHCs), idiopathic Parkinson's disease (iPD) patients, *LRRK2*‐PD patients, and *GBA*‐PD patients after *P. aeruginosa* treatment. (A) HLA‐DR expression in T cells (effect of interaction *P* = 0.9805, treatment *P* = 0.1262, cohort *P* = 0.0031). (B) HLA‐DR expression in classical monocytes (effect of interaction *P* = 0.9010, treatment *P* = 0.9867, cohort *P* = 0.0001). (C) HLA‐DR expression in intermediate monocytes (effect of interaction *P* = 0.9218, treatment *P* = 0.0961, cohort *P* = 0.0498). (D) HLA‐DR expression in nonclassical monocytes (effect of interaction *P* = 0.2178, treatment *P* = 0.0152, cohort *P* = 0.0047). (E) HLA‐DR expression in CD3^−^/CD14^−^/CD16^−^ cells (effect of interaction *P* = 0.5764, treatment *P* = 0.0579, cohort *P* = 0.0010). Bars represent mean ± standard error of the mean. NHCs, n = 24; iPD, n = 33; *LRRK2‐PD*, n = 21; *GBA‐*PD, n = 17. Each symbol represents the measurement from a single individual. The results in A–E were analyzed using two‐way analysis of variance with Tukey's corrections for multiple comparisons. Only comparisons within treatment and comparisons within cohorts across treatment are shown. Statistically significant differences defined by *P* < 0.05 are shown. [Color figure can be viewed at wileyonlinelibrary.com]

Next, we sought to investigate whether other metrics of immune cell activation, such as antigen presentation in response to pathogen exposure, were affected by *LRRK2* or *GBA1* mutations and treatment with MLi‐2 or with NCG. Expression of major histocompatibility complex (MHC) class II has been found to be increased in postmortem brain tissue from PD patients,^40^ suggesting a potential link between PD and dysregulated antigen presentation in immune cells. Therefore, we chose to evaluate the surface expression of the ‐DR isotype of human leukocyte antigen (HLA), which is a component of MHC‐II and functions to present antigens for recognition by other immune cells.^41^ To accomplish this, we stained cells with antibody‐fluorophore conjugates for HLA‐DR as a metric for antigen presentation. In unstimulated classical and intermediate monocytes, antigen presentation was not significantly different across cohorts (Fig. S4A,B). In *P. aeruginosa*‐(PA) stimulated samples, we observed that T cells displayed no significant differences in HLA‐DR expression across cohorts with no treatment effect of MLi‐2 or NCG (Fig. 2A). Classical monocytes from *LRRK2*‐PD patients showed significantly increased HLA‐DR expression relative to iPD in the MLi‐2‐treated condition, however, no differences were observed in *P. aeruginosa* only or NCG conditions (Fig. 2B). Intermediate monocytes showed similar levels of antigen presentation across cohorts in all treatment conditions (Fig. 2C). However, in nonclassical monocytes, there was a significant effect of treatment on antigen presentation (treatment effect *P* = 0.0015), and NCG treatment induced greater HLA‐DR expression in *LRRK2*‐PD samples than PA alone or MLi‐2 (Fig. 2D). We observed that CD3^−^/CD14^−^/CD16^−^ cells from iPD patients showed decreased HLA‐DR expression relative to HCs in the drug‐free condition (Fig. 2E). Overall, these data suggest that antigen presentation in response to *P. aeruginosa* can be modulated by enhanced GCase activation.

### LRRK2 Kinase Inhibition and GCase Activation Can Separately Modulate pRab10 Expression in *P. Aeruginosa*‐Treated PBMCs

The surface expression of MHC‐II relies on effective trafficking through the endocytic pathway, moving from the endoplasmic reticulum to early endosomes to late endosomal/lysosomal compartments.^42^ LRRK2 kinase activity has been shown to regulate endosomal/lysosomal maturation, with pharmacologic inhibition of LRRK2 kinase serving to rescue endosomal maturation and mitigate neurodegeneration in a mouse model of iPD.^43^ Therefore, altered surface levels of HLA‐DR in stimulated PBMCs could be influenced by differences in LRRK2 kinase activity. Our group has previously demonstrated that LRRK2 regulates surface expression of MHC‐II through lysosomal tubule formation.^44^ Given these findings and the emergence of LRRK2 inhibitors as potential disease modifying therapies,^45^, ^46^ we sought to evaluate the expression of pRab10, a bona fide substrate of LRRK2 kinase,^47^ in stimulated PBMCs as a reporter of LRRK2 kinase activity.

**FIG. 3:**
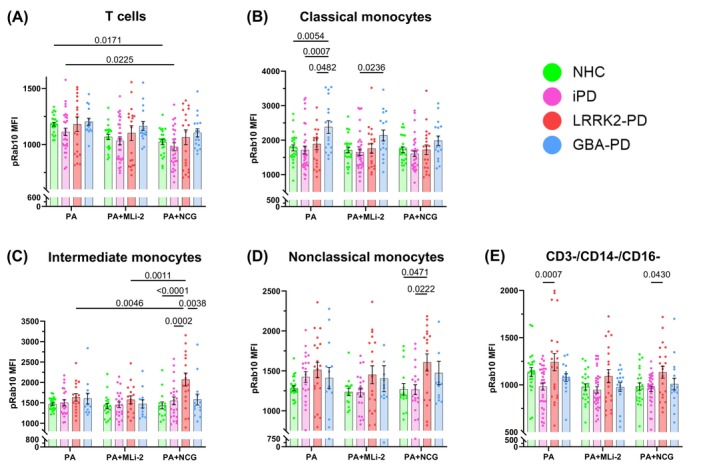
Stimulation‐dependent pRab10 expression is modulated by *LRRK2*‐Parkinson's disease (PD) and glucocerebrosidase (GCase) activity. Bar graphs overlaid with scatter plots showing expression of phosphorylated Rab10 (pRab10) in peripheral blood mononuclear cells (PBMCs) from neurologically healthy controls (NHCs), idiopathic PD (iPD) patients, *LRRK2‐*PD patients, and *GBA*‐PD patients after *Pseudomonas aeruginosa* (*P. aeruginosa*) treatment. (A) pRab10 mean fluorescence intensity (MFI) in CD3^+^ T cells (effect of interaction *P* = 0.9911, treatment *P* = 0.0002, cohort *P* = 0.0077). (B) pRab10 MFI in classical monocytes (effect of interaction *P* = 0.9191, treatment *P* = 0.0952, cohort *P* < 0.0001). (C) pRab10 MFI in intermediate monocytes (effect of interaction *P* = 0.1142, treatment *P* = 0.0385, cohort *P* = 0.0005). (D) pRab10 MFI in nonclassical monocytes (effect of interaction *P* = 0.7951, treatment *P* = 0.3945, cohort *P* = 0.0009). (E) pRab10 MFI in CD3^−^/CD14^−^/CD16^−^ cells (effect of interaction *P* = 0.7917, treatment *P* = 0.0050, cohort *P* < 0.0001). Bars represent mean ± standard error of the mean. NHCs, n = 24; iPD, n = 33; *LRRK2‐PD*, n = 21; *GBA‐*PD, n = 17. Each symbol represents the measurement from a single individual. The results in A–E were analyzed using two‐way analysis of variance with Tukey's corrections for multiple comparisons. Only comparisons within treatment and comparisons within cohorts across treatment are shown. Statistically significant differences defined by *P* < 0.05 are shown. [Color figure can be viewed at wileyonlinelibrary.com]

In unstimulated monocytes, we observed that baseline expression of pRab10 was similar across all cohorts (Fig. S4). Following *P. aeruginosa* exposure, T‐cell expression of pRab10 was similar across all cohorts, however, NCG treatment caused a reduction in pRab10 in NHCs and iPD (treatment effect *P* = 0.0002) (Fig. 3A). Moreover, classical monocyte pRab10 expression was significantly increased in *GBA*‐PD relative to all other cohorts (cohort effect *P* < 0.0001) (Fig. 3B). In intermediate monocytes stimulated with *P. aeruginosa* alone, the addition of NCG elicited increased pRab10 expression in *LRRK2*‐PD relative to other cohorts (cohort effect *P* = 0.0005; treatment effect *P* < 0.0385) (Fig. 3C). However, in the NCG‐treated group, we observed significantly increased pRab10 expression in *LRRK2‐*PD relative to all other cohorts (NHC and iPD groups treatment effect *P* = 0.0018). In nonclassical monocytes treated with NCG, *LRRK2*‐PD samples exhibited increased pRab10 expression compared to NHC and iPD groups (cohort effect *P* = 0.0009) (Fig. 3D). We also observed that CD3^−^/CD14^−^/CD16^−^ cells from *LRRK2‐*PD patients showed increased pRab10 expression relative to iPD in PA alone and NCG‐treated conditions (cohort effect *P* < 0.0001) (Fig. 3E). Together, these results suggest that enhanced GCase activation modulates LRRK2 kinase activity as measured by pRab10 levels in immune cells within the context of *P. aeruginosa* stimulation, primarily in intermediate monocytes.

### Stimulation‐Dependent LRRK2 Expression Is Reduced by MLi‐2 and NCG

**FIG. 4:**
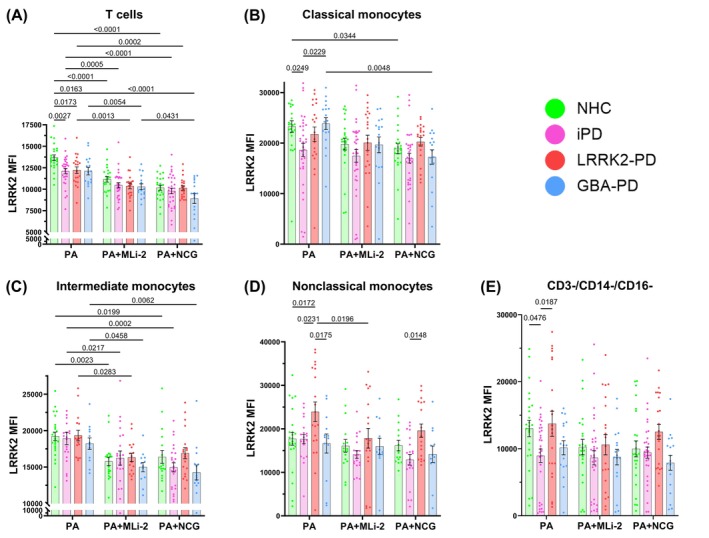
Effects of LRRK2 kinase inhibition and glucocerebrosidase (GCase) activation on stimulation‐dependent expression of LRRK2. Bar graphs overlaid with scatter plots showing expression of LRRK2 in peripheral blood mononuclear cells (PBMCs) from neurologically healthy controls (NHCs), idiopathic Parkinson's disease (iPD) patients, *LRRK2‐PD* patients, and *GBA*‐PD patients after *Pseudomonas aeruginosa (P. aeruginosa)* treatment. (A) LRRK2 mean fluorescence intensity (MFI) in CD3^+^ T cells (effect of interaction *P* = 0.3118, treatment *P* < 0.0001, cohort *P* = 0.0004). (B) LRRK2 MFI in classical monocytes (effect of interaction *P* = 0.5111, treatment *P* = 0.0004, cohort *P* = 0.0029). (C) LRRK2 MFI in intermediate monocytes (effect of interaction *P* = 0.8221, treatment *P* < 0.0001, cohort *P* = 0.0983). (D) LRRK2 MFI in nonclassical monocytes (effect of interaction *P* = 0.6949, treatment *P* = 0.0091, cohort *P* = 0.0001). (E) LRRK2 MFI in CD3^−^/CD14^−^/CD16^−^ cells (effect of interaction *P* = 0.6618, treatment *P* = 0.0699, cohort *p* = 0.0012). Bars represent mean ± standard error of the mean. NHCs, n = 24; iPD, n = 33; *LRRK2‐PD*, n = 21; *GBA‐*PD, n = 17. Each symbol represents the measurement from a single individual. The results in A–E were analyzed using two‐way analysis of variance with Tukey's corrections for multiple comparisons. Only comparisons within treatment and comparisons within cohorts across treatment are shown. Statistically significant differences defined by *P* < 0.05 are shown. [Color figure can be viewed at wileyonlinelibrary.com]

LRRK2 kinase activity and expression of LRRK2 is upregulated in immune cells in response to immunogenic stimuli.^34^, ^48^, ^49^ Some data suggest that the increased kinase activity of mutant LRRK2 potentially plays a key role in stabilizing its own protein conformation,^50^ therefore, contributing to increased LRRK2 levels. Therefore, we sought to explore the effects of pharmacologic modulation of LRRK2 kinase and GCase activity on stimulation‐dependent LRRK2 expression. In unstimulated PBMCs, we observed that baseline levels of LRRK2 protein were similar across all cohorts (Fig. S4). However, after *P. aeruginosa* stimulation, we observed that T cells from NHCs displayed increased LRRK2 expression relative to all other cohorts, with both MLi‐2 and NCG treatment ablating these differences (cohort effect *P* = *0*.0004; treatment effect *P* < 0.0001) (Fig. 4A). LRRK2 expression in classical monocytes was reduced in iPD relative to NHC and *GBA*‐PD, but both MLi‐2 and NCG treatments ablated these differences (cohort effect *P* = 0.0029; treatment effect *P* = 0.0004) (Fig. 4B). Intermediate monocytes showed similar expression of LRRK2 across cohorts in PA alone, but interestingly, both MLi‐2 and NCG caused significant reductions in LRRK2 expression (treatment effect *P* < 0.0001) (Fig. 4C). Stimulated nonclassical monocytes from *LRRK2‐*PD patients showed greater LRRK2 expression than NHC and *GBA*‐PD samples, and MLi‐2 resulted in the ablation of across‐cohort differences (cohort effect *P* = 0.0001; treatment effect *P* = 0.0091) (Fig. 4D). Stimulation‐dependent LRRK2 expression in CD3^−^/CD14^−^/CD16^−^ was reduced in iPD relative to NHC and *LRRK2‐*PD samples (cohort effect *P* = 0.0012), while no differences were observed across cohorts in the MLi‐2 and NCG treatment conditions (Fig. 4E). These results overall indicate that regulation of LRRK2 protein levels in response to *P. aeruginosa* is disrupted in PBMC subtypes. Additionally, MLi‐2 and NCG treatment both serve to attenuate LRRK2 levels in subsets of stimulated immune cells (summary of findings described in Fig. S7).

## Discussion

Two of the most commonly mutated genes in familial PD, *LRRK2* and *GBA1*, converge on immune and lysosomal function,^51^ but the precise mechanisms by which these mutations contribute to PD pathogenesis remain incompletely understood. Here, we developed a protocol to capture the immune response to bacterial pathogens in plated PBMCs using live *P. aeruginosa* and applied this protocol to interrogate the effects of PD‐associated mutations in *LRRK2* and *GBA1* on peripheral blood immune cell effector functions. Our methodology revealed differences in pathogen‐evoked cytokine secretion, antigen presentation, LRRK2 kinase activity, and LRRK2 protein expression based on disease and mutation status. Our findings reveal a cell type‐specific effect of GCase activity on HLA‐DR expression in response to bacterial stimulation, with NCG enhancing antigen presentation in *LRRK2*‐PD intermediate and nonclassical monocytes. Furthermore, NCG decreased pRab10 levels in T cells, whereas both MLi‐2 and NCG reduced LRRK2 protein levels in immune cells after exposure to *P. aeruginosa*. Our findings suggest that the application of a chaperone protein, which enhances GCase activity can modulate LRRK2 protein expression and kinase activity in PBMCs, an important consideration for future therapeutic interventions.

**FIG. 5:**
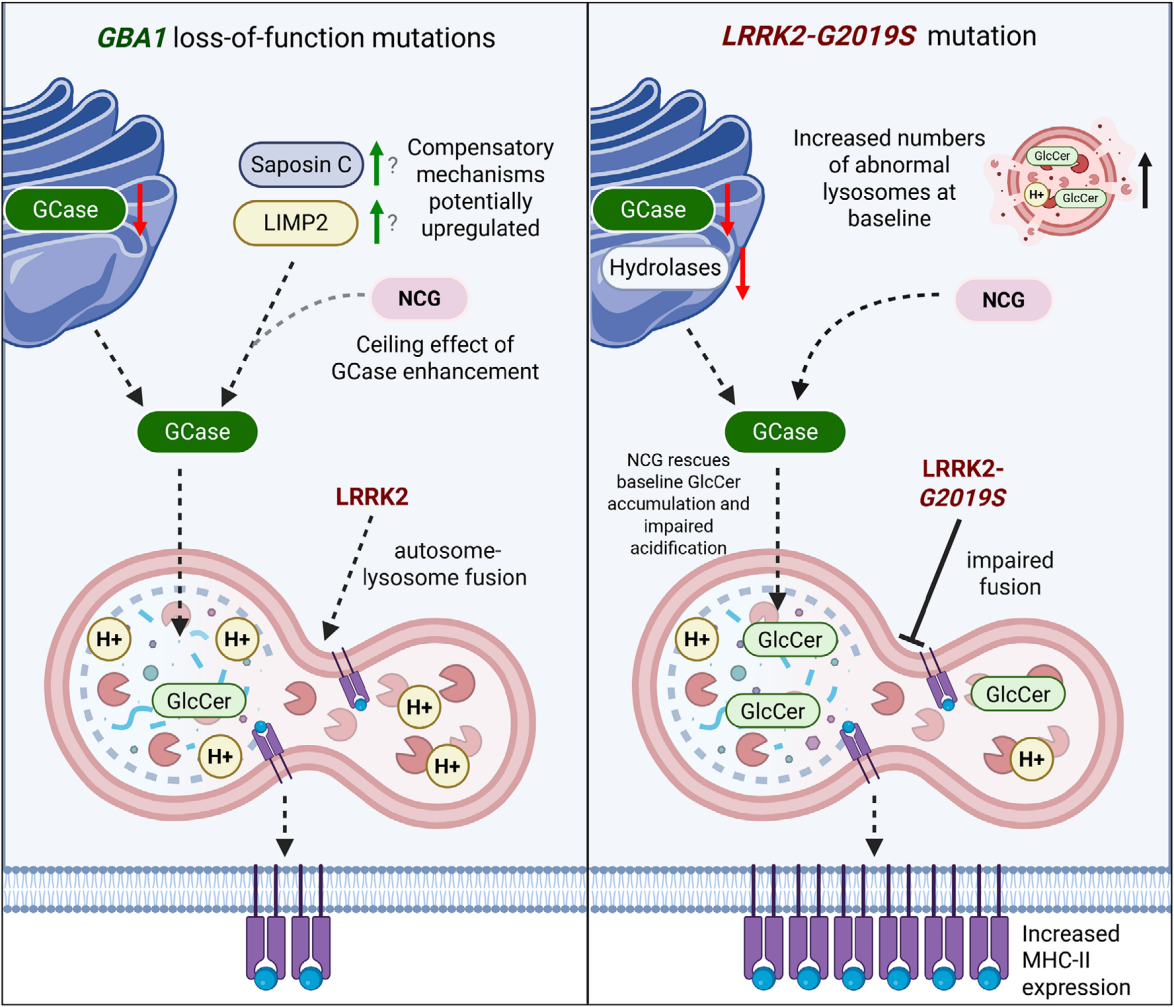
Proposed model for NCGC00188758 (NCG) effects at the lysosome in *GBA1* and *LRRK2* mutant immune cells. *GBA1* mutant cells express decreased levels of glucocerebrosidase (GCase) at baseline. In response to immune stimulation, compensatory mechanisms that enhance/support GCase function are upregulated, contributing to a ceiling effect where NCG has limited ability to further increase GCase activity. In *LRRK2* mutant cells, lysosomal defects are more widespread and compensatory mechanisms may be less effective. This leads to lysosomal buildup of glucosylceramide (GlcCer), impaired acidification, and an accumulation of dysfunctional lysosomes. NCG improves GCase trafficking in these cells, partially rescuing lysosomal defects, and contributes to increased major histocompatibility complex class II (MHC‐II) processing and surface expression. [Color figure can be viewed at wileyonlinelibrary.com]

Other groups have identified that ex vivo systems are powerful tools for studying immune responses in aging and disease, providing significant advantages in terms of reproducibility and the level of environmental control that can be achieved.^52^, ^53^ Our findings demonstrate that PBMCs from *GBA*‐PD individuals exhibit enhanced cytokine secretion in response to *P. aeruginosa* exposure relative to NHCs and iPD individuals. Others have shown that excessive inflammation in PD models can exacerbate α‐synuclein pathology and accelerate dopaminergic neuronal death.^54^ Our results from these ex vivo stimulation studies are consistent with previously findings from Miliukhina et al^55^ who observed increased circulating levels of IL‐1β in GBA‐PD patients compared to both sporadic PD and controls. In addition, increased baseline levels of CCL18^56^ and IL‐8^57^ have been associated with *GBA* mutations. IL‐1β secretion from PBMCs was not affected by MLi‐2 or NCG treatment, which may indicate that inflammasome activation in response to *P. aeruginosa* occurs independently of LRRK2 kinase and GCase activity. Intriguingly, stimulation‐evoked IL‐2 secretion from PBMCs was not significantly different across patient cohorts in the *P. aeruginosa* alone and MLi‐2‐treated conditions, but NCG treatment elicited significantly increased IL‐2 secretion in *GBA*‐PD samples compared to all other cohorts. IL‐2 is primarily secreted by activated T cells,^58^, ^59^ therefore, these results may indicate that GCase is particularly important for immune activation in T cells. One possible explanation for these results is that immune cells heterozygous for a loss‐of‐function *GBA1* mutation could have reduced GCase activity at baseline and may use compensatory mechanisms (Fig. 5). For example, it was recently reported that *GBA1‐*mutant neurons upregulate calcium‐buffering properties as a compensatory protective mechanism against endoplasmic reticulum (ER) stress.^60^ In immune cells, short term enhancement of GCase activity with NCG treatment may work synergistically to contribute to increased IL‐2 secretion. Therefore, it will be necessary for therapeutic strategies aimed at rescuing GCase activity in *GBA*‐PD to take into consideration these potential stimulation‐evoked effects on the peripheral blood immune response.

It is surprising that the stimulation‐based response to PA appears similar between iPD individuals and controls in terms of cytokine secretion and antigen presentation. This is somewhat at odds with previous reports that baseline markers of inflammation such as the neutrophil‐to‐lymphocyte ratio, are significantly increased in both iPD and *GBA‐*PD individuals compared to NHCs.^6^ In our study, immune stimulation was required to observe increased cytokine secretion in *GBA*‐PD, suggesting that variability in baseline levels of inflammation may make it difficult to demonstrate subtle immune dysfunction. It has previously been reported that *LRRK2‐*PD individuals showed a similar neutrophil‐to‐lymphocyte ration and baseline level of inflammation to control individuals.^6^ Similarly, others have shown that IL‐10 and IL‐12p40 serum levels are greater in iPD relative to *LRRK2*‐PD.^61^ Moreover, *LRRK2*‐PD has been associated with higher levels of platelet‐derived growth factor, vascular endothelial growth factor, and IL‐8 compared to iPD,^21^ and both forms of PD show increased levels of the proinflammatory marker fatty‐acid binding protein relative to NHCs.^61^ In the context of the literature, which suggests that baseline levels of inflammation are dysregulated in iPD, our findings suggest that iPD does not significantly impact the inducible ex vivo immune response to *P. aeruginosa*.

Concurrently, we observed that nonclassical monocytes from *LRRK2‐*PD individuals showed increased antigen presentation relative to iPD and *GBA‐*PD when treated with the GCase activity enhancer NCG. A key step in MHC‐II processing is the transport of peptide‐loaded MHC‐II molecules from endocytic vesicles to the plasma membrane, a step that is facilitated by lysosomal tubule formation (LTF).^44^, ^62^ We recently showed that increased LRRK2 kinase activity was associated with enhanced LTF and increased MHC‐II trafficking to the plasma membrane in a mTOR‐dependent manner in *Lrrk2‐G2019S* mice.^44^ The literature suggests that LRRK2 interacts with MHC‐II expression in an age‐dependent manner. Specifically, LRRK2 kinase inhibition in young mice causes reduced MHC‐II expression,^44^, ^63^ whereas gain‐of‐function kinase mutations in aged animals also reduce MHC‐II expression and are linked to immune exhaustion.^64^ Given that the average age of PD participants was greater than 64, it would be reasonable to expect that MLi‐2 would enhance immune function in older or “aged” *LRRK2‐G2019S* PD patients. However, this was not observed in monocytes and T cells. Instead, it was NCG treatment that caused increased HLA‐DR levels in *LRRK2*‐PD nonclassical monocytes. There are a few considerations that might help to explain this finding. LRRK2 kinase activity increases in response to immune activation,^34^, ^49^, ^65^ but it is possible that a ceiling effect was reached. This would mean that both the changes in MHC‐II expression because of LRRK2‐mediated LTF and the gain‐of‐function kinase activity because of the *G2019S* mutation could be masked. In support of this, we observed that in intermediate monocytes, NCG induces a *LRRK2‐*PD‐specific increase in pRab10 and MHC‐II. This could speak to the fact that increased GCase activation may enhance certain cellular functions that augment MHC‐II expression, such as lysosomal clearance, which are dysfunctional in *LRRK2*‐PD immune cells and are incompletely rescued by MLi‐2. It is possible that compensatory mechanisms that support GCase function are upregulated in *GBA1* mutant cells, and this may include increased expression of saposin C and lysosomal integral membrane protein‐2 (LIMP2), which are known to enhance GCase activity.^66^, ^67^ LIMP2 has previously been reported to be increased in *GBA*‐PD PBMCs.^68^ Therefore, the effect of NCG could be masked by a ceiling effect in stimulated immune cells from *GBA1* mutation carriers. Moreover, the *G2019S*‐*LRRK2* mutation is associated with impaired lysosomal acidification and increased numbers of dysfunctional lysosomes.^69^ Our results are consistent with a hypothetical model where NCG partially rescues baseline dysregulation present in *LRRK2*‐PD cells, enhancing acidification and clearance of glucosylceramide, which contributes to greater downstream MHC‐II expression (Fig. 5). However, these results should be interpreted with caution given that our experimental conditions did not reveal a dysfunctional condition at baseline in *LRRK2* mutant cells.

GCase activity in plated monocytes has been previously quantified ex vivo, with intermediate monocytes exhibiting lower GCase activity relative to classical and nonclassical monocytes.^70^ In the present study, the limited cell numbers available per sample precluded us from evaluating GCase activity in a live cell flow cytometry panel. Therefore, it will be valuable for future studies to investigate potential cell‐type specific differences in GCase expression and variable response to GCase modulation across immune cell subtypes in the context of stimulation‐evoked responses. Interestingly, MLi‐2 and NCG both caused reduced antigen presentation in CD3^−^ non‐monocytes. This suggests that both LRRK2 and GCase activity regulate surface expression of HLA‐DR after stimulation, but their role in supporting immune activation or processing of HLA‐DR may vary depending on immune cell type.

Furthermore, we demonstrate that LRRK2 kinase activation measured by levels of pRab10 after immune stimulation is modulated by the GCase activator, NCGC00188758. We observed that NCG treatment significantly increased pRab10 in *LRRK2*‐PD intermediate monocytes compared to both MLi‐2 and *P. aeruginosa* alone. pRab10 is known to maintain lysosomal homeostasis and fulfill roles in lysosomal stress response pathways,^71^, ^72^ therefore, our results imply that GCase may support endolysosomal function through regulation of pathways also regulated by LRRK2. LRRK2 has previously been reported to negatively regulate GCase activity,^73^ but our results suggest that GCase may positively regulate mutant LRRK2 kinase activity in specific monocyte subtypes. Another possibility is that these effects are mediated by interactions with phosphatases, such as PPM1H, which dephosphorylate Rab proteins.^74^ Dephosphorylation of Rab10 by PPM1H requires correct localization of the phosphatase at the mother centriole,^75^ and GCase function has been implicated in the regulation of organelle trafficking and positioning.^76^ Further research is necessary to evaluate interactions between GCase and other regulators of Rab10 phosphorylation to determine the mechanism by which GCase modulates these pathways in the context of immune stimulation.

Notably, our findings suggest that treatment with NCG can modulate LRRK2 expression in immune cells. NCG is a chaperone protein that enhances folding of GCase and improves protein translocation, thereby increasing GCase activity.^77^ Our results demonstrate that NCG caused significant reductions in LRRK2 expression in T cells and monocytes that had been exposed to *P. aeruginosa*. Given that LRRK2 expression is generally upregulated in immune cells in response to stimulation,^9^, ^65^, ^78^ our results may indicate that treatment with NCG mitigates stimulation‐dependent increases in LRRK2 expression. Chronic MLi‐2 treatment has previously been shown to cause a reduction in LRRK2 total protein levels in exosomes from non‐human primates.^79^ Here, we observed that MLi‐2 treatment was effective in abrogating across‐cohort differences in LRRK2 protein expression in T cells, intermediate monocytes, and nonclassical monocytes. Interestingly, NCG treatment showed similar effects to MLi‐2, with NCG also reducing LRRK2 levels and largely removing cohort differences. Therefore, our results in peripheral blood immune cells complement prior work by Pang et al^80^ who reported that LRRK2 mutation is associated with reduced GCase activity and LRRK2 kinase inhibition can modulate GCase activity.

Our results suggest that LRRK2 levels are generally suppressed by both MLi‐2 and NCG in individuals with and without mutations in *LRRK2* or *GBA1*, which may indicate a nonspecific effect of these pharmacological interventions. MLi‐2 is considered a non‐selective LRRK2 inhibitor that reduces LRRK2 kinase activity regardless of mutation status,^81^ and it has some off‐target effects for inhibiting kinases besides LRRK2 such as CLK2, CLK4, MAP3K14, MAP3K5, and TTK.^82^ Therefore, further study with a compound like EB‐42168, which is a specific inhibitor *G2019S* mutant LRRK2^81^ would be helpful in demonstrating mutation‐specific effects given that *LRRK2*‐PD patients are generally heterozygous. A limitation of this study is that the *GBA*‐PD cohort possessed a mixture of pathogenic variants that may have different effects on GCase activity. For example, the *N370S* allele is reported to show approximately 32% of the residual function of the wild‐type allele, whereas the *L444P* variant shows 23% residual function compared to wild‐type.^83^ These baseline differences may have contributed to variability, and it will be valuable to conduct future studies that are powered to detect differences between these *GBA1* variants in the context of immune activation. NCG is purported to act as a small‐molecule chaperone protein for misfolded GCase, enabling stabilization of the correct protein conformation and improving trafficking to its target destination of the lysosome.^84^ This chaperone is effective in modifying activity of mutant *N370S* GCase,^84^ but a key consideration for future research will be to determine if NCG shows variable effectiveness across other genetic variants as this remains a gap in the current literature. Our study did not analyze asymptomatic carriers of *LRRK2* and *GBA1* mutations, therefore, the inclusion of non‐manifesting mutation carriers will be important for future studies to isolate the contributions of these mutations independently of PD status and potentially to enable timely interventions to delay or arrest progression to motor PD in mutation carriers.

Because of the limited availability of human PBMC samples, we were unable to conduct a live cell flow cytometry panel to measure GCase enzymatic activity after stimulation. Follow up studies that include this approach or lipid quantification of GCase protein function^85^, ^86^ to evaluate degradative capacity and the degree to which NCG treatment modifies GCase activity after bacteria stimulation will be informative. In follow up studies, it will be interesting to characterize the effects of *LRRK2* and *GBA1* on immune response in a broader range of disease‐relevant peripheral immune cell subtypes such as B cells and natural killer cells. Moreover, it will be informative to test the combination of MLi‐2 and NCG simultaneously with stimulation to determine if these compounds have synergistic effects in affecting cytokine secretion and antigen presentation. It is important to acknowledge that there is a significant variability to these responses, only some of which can be attributed to the expected variability with human samples. Although effort was taken to ensure that each sample experienced an equal bacterial load during stimulation, slight differences in the absolute number of PA units may have arisen. Given that the doubling time of PA can be as low as 30 minutes in rich broth,^87^, ^88^ the long duration time selected in this study may have amplified minor differences in initial bacterial loading. That is, the highly technical nature of the experimental protocol used here may have contributed to the observed variability. We tested a single stimulation duration to preserve the limited number of samples available. This duration was selected based on prior work by others using PBMC stimulations with *P. aeruginosa* ^32^ and our own preliminary optimization experiments. However, peak cytokine production does not occur at a singular uniform time point and instead varies with the type of cytokine and stimulus.^89^ Therefore, it would be valuable for future studies to investigate treatment durations shorter than 24 hours, which may mitigate variability in live bacterial reproduction speed and provide further insight into the kinetics of cytokine secretion in the context of LRRK2 and GCase mutations or manipulation.

Our findings are in agreement with those of other groups who report that *LRRK2* and *GBA1* interact with one another at the lysosome,^51^, ^73^, ^80^ but larger scope analyses such as RNA‐sequencing and proteomics will help define the exact molecular mechanisms by which these proteins regulate each other. Moreover, the results presented here represent the immune response to a single pathogen, *P. aeruginosa*, which is gram‐negative and typically extracellular.^90^ In future studies, it will be necessary to characterize how *LRRK2* and *GBA1* mutations affect the immune response to an expanded range of pathogens, including gram‐positive bacteria, mycobacterium, and viruses. Given that a wide range of immune insults, including chronic gut inflammation^91^, ^92^ and severe influenza infections,^25^, ^93^ have been linked to increased PD risk, further investigations with these pathogens may reveal pathogen‐specific interactions with *LRRK2* and *GBA1*.

In summary, the significance of our findings is three‐fold. First, we have successfully developed and optimized methods to stimulate human PBMCs with pathogens ex vivo to assess peripheral immune dysfunction traits that could help in stratifying LRRK2‐ or GBA‐mutation carriers for clinical trials. Second, we have discovered that PD‐linked mutations in *LRRK2* and *GBA1* are associated with differential peripheral blood immune responses to pathogens, including alterations in cytokine secretion, antigen presentation, and LRRK2 kinase activity. Finally, our findings suggest that pharmacologic interventions that modulate LRRK2 kinase and GCase activity converge on similar pathways in endolysosomal regulation in the peripheral blood immune response to bacterial pathogens. The significance of our findings underscores the importance of the immune‐mediated response in the environmental risk for PD in genetically predisposed individuals. The variability in the ex vivo responses to stimulation may be related to the heterogeneous gene‐by‐environment interaction outcomes for different individuals, but significant further study is warranted to determine the relationship between immune dysfunction and clinical progression of PD. Additional research is required to determine the mechanisms by which GCase and LRRK2 interact with one another and influence downstream immune function, with particular interest paid toward how pathways involving these proteins may intersect at the lysosome. Because of the increasing evidence that immune dysregulation is a contributing factor in the pathophysiology of PD, continued investigations into the connections between PD‐linked mutations and peripheral blood immune cell dysfunction will provide insight into mechanisms that modify risk and/or progression of PD.

## Author Roles

(1) Research Project: A. Conception, B. Organization, C. Execution; (2) Statistical Analysis: A. Design, B. Execution, C. Review and Critique; (3) Manuscript: A. Writing of the First Draft, B. Review and Critique.

J.R.M.: 1A, 1B, 1C, 2A, 2B, 2C, 3A, 3B.

H.A.S.: 1C, 3B.

A.M.T.: 1C, 3B.

J.A‐L.: 1C, 3B.

A.G.: 1C, 3B.

L.H.: 1A, 1B, 1C, 2A, 2C, 3B.

N.D.: 1A, 1B, 1C, 2A, 2C, 3B.

R.L.W.: 1A, 1B, 1C, 2A, 2C, 3B.

M.G.T.: 1A, 1B, 1C, 2A, 2C, 3B.

## Supporting information


Figure S1.

Figure S2.

Figure S3.

Figure S4.

Figure S5.

Figure S6.

Figure S7.

Table S1.

## Data Availability

The data that support the findings of this study are available from the corresponding author upon reasonable request.
