# Supplementary material for: Parkinson's‐Linked LRRK2 and GBA1 Mutations Modulate the Peripheral Immune Response to Pseudomonas aeruginosa
Source: Mov Disord. 2025 Nov 19;41(3):651–66. doi: 10.1002/mds.70123 (PMC13022586; doi:10.1002/mds.70123)
Supplement: Supplementary file 1 — Figure S1. [file MDS-41-651-s005.pptx]

## Slide 1
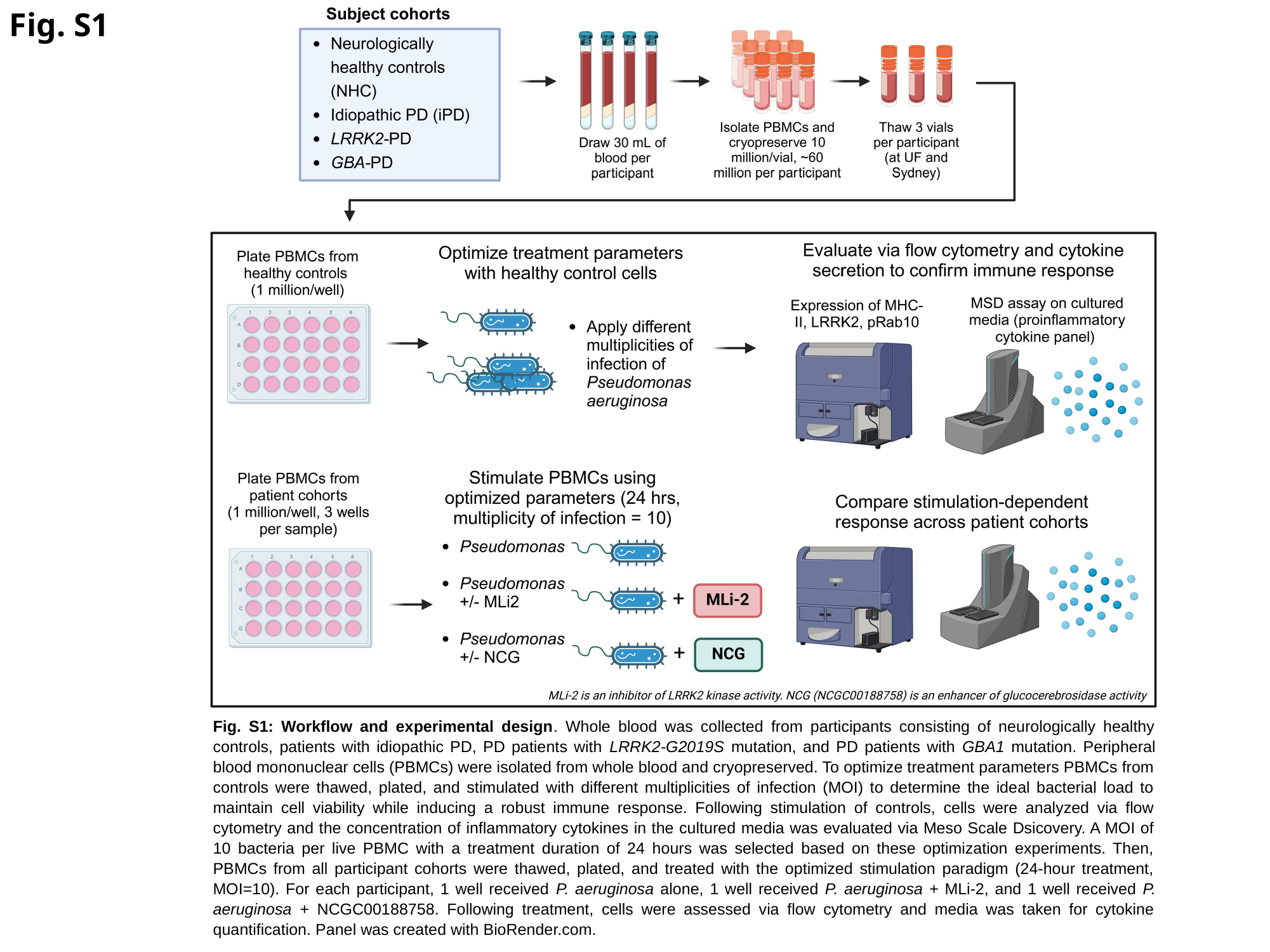

Fig. S1
Fig. S1: Workflow and experimental design. Whole blood was collected from participants consisting of neurologically healthy controls, patients with idiopathic PD, PD patients with LRRK2-G2019S mutation, and PD patients with GBA1 mutation. Peripheral blood mononuclear cells (PBMCs) were isolated from whole blood and cryopreserved. To optimize treatment parameters PBMCs from controls were thawed, plated, and stimulated with different multiplicities of infection (MOI) to determine the ideal bacterial load to maintain cell viability while inducing a robust immune response. Following stimulation of controls, cells were analyzed via flow cytometry and the concentration of inflammatory cytokines in the cultured media was evaluated via Meso Scale Dsicovery. A MOI of 10 bacteria per live PBMC with a treatment duration of 24 hours was selected based on these optimization experiments. Then, PBMCs from all participant cohorts were thawed, plated, and treated with the optimized stimulation paradigm (24-hour treatment, MOI=10). For each participant, 1 well received P. aeruginosa alone, 1 well received P. aeruginosa + MLi-2, and 1 well received P. aeruginosa + NCGC00188758. Following treatment, cells were assessed via flow cytometry and media was taken for cytokine quantification. Panel was created with BioRender.com.
