# Supplementary material for: Parkinson's‐Linked LRRK2 and GBA1 Mutations Modulate the Peripheral Immune Response to Pseudomonas aeruginosa
Source: Mov Disord. 2025 Nov 19;41(3):651–66. doi: 10.1002/mds.70123 (PMC13022586; doi:10.1002/mds.70123)
Supplement: Supplementary file 2 — Figure S2. [file MDS-41-651-s003.pptx]

## Slide 1
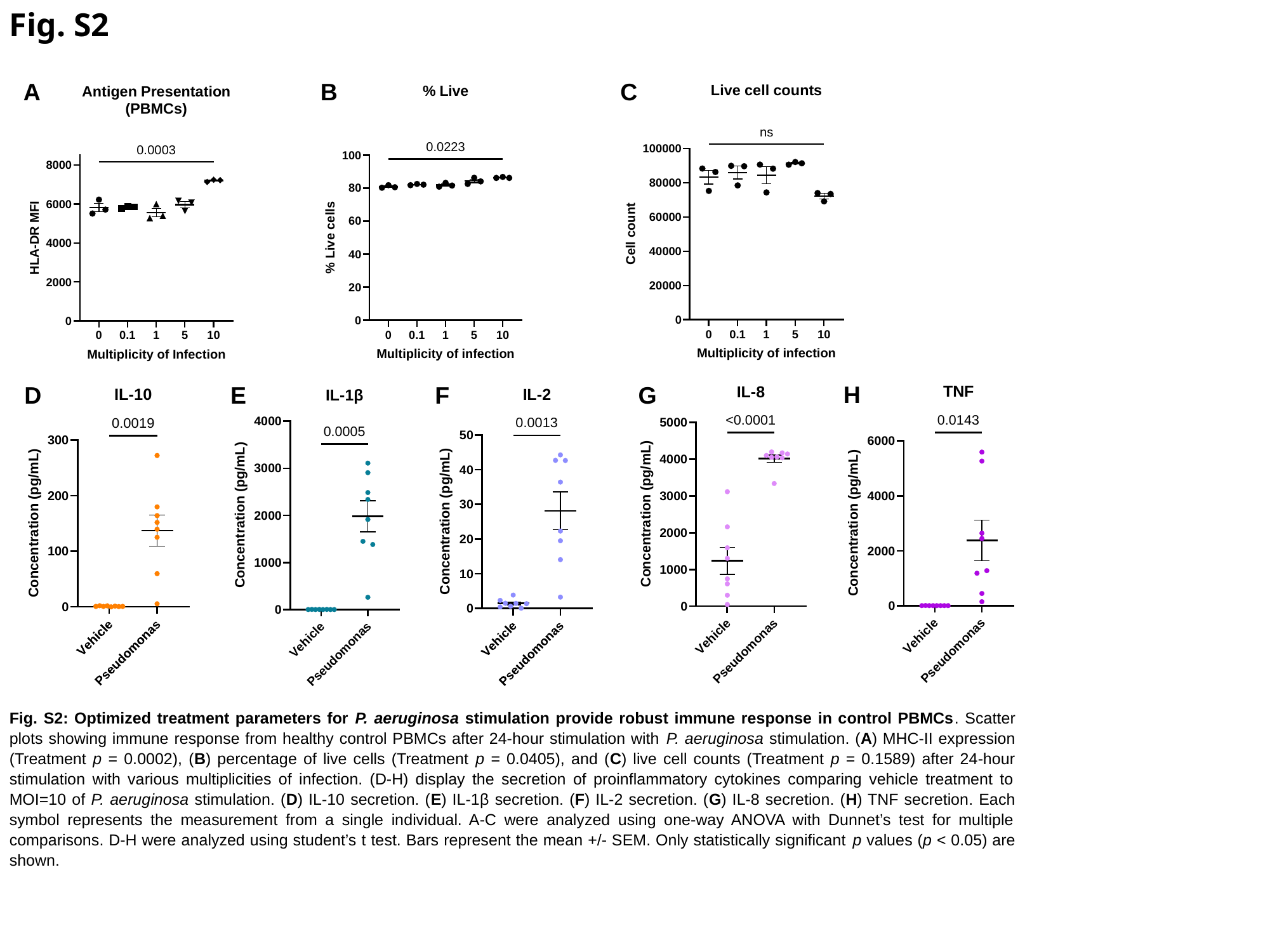

Fig. S2
A
B
C
H
D
E
G
F
Fig. S2: Optimized treatment parameters for P. aeruginosa stimulation provide robust immune response in control PBMCs. Scatter plots showing immune response from healthy control PBMCs after 24-hour stimulation with P. aeruginosa stimulation. (A) MHC-II expression (Treatment p = 0.0002), (B) percentage of live cells (Treatment p = 0.0405), and (C) live cell counts (Treatment p = 0.1589) after 24-hour stimulation with various multiplicities of infection. (D-H) display the secretion of proinflammatory cytokines comparing vehicle treatment to MOI=10 of P. aeruginosa stimulation. (D) IL-10 secretion. (E) IL-1β secretion. (F) IL-2 secretion. (G) IL-8 secretion. (H) TNF secretion. Each symbol represents the measurement from a single individual. A-C were analyzed using one-way ANOVA with Dunnet’s test for multiple comparisons. D-H were analyzed using student’s t test. Bars represent the mean +/- SEM. Only statistically significant p values (p < 0.05) are shown.
