# Supplementary material for: Parkinson's‐Linked LRRK2 and GBA1 Mutations Modulate the Peripheral Immune Response to Pseudomonas aeruginosa
Source: Mov Disord. 2025 Nov 19;41(3):651–66. doi: 10.1002/mds.70123 (PMC13022586; doi:10.1002/mds.70123)
Supplement: Supplementary file 3 — Figure S3. [file MDS-41-651-s008.pptx]

## Slide 1
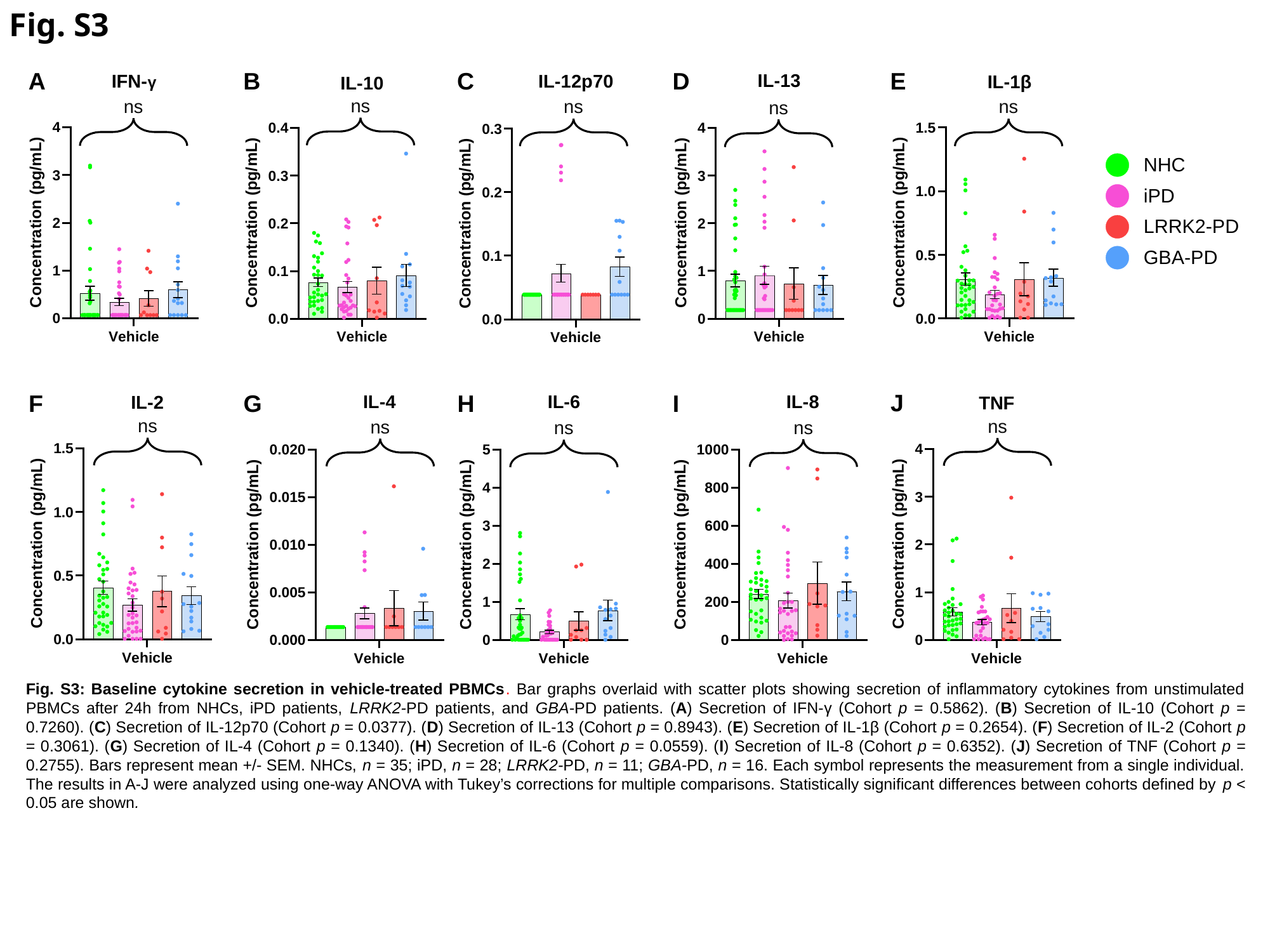

Fig. S3
E
A
B
D
C
ns
ns
ns
ns
ns
J
F
G
I
H
ns
ns
ns
ns
ns
Fig. S3: Baseline cytokine secretion in vehicle-treated PBMCs. Bar graphs overlaid with scatter plots showing secretion of inflammatory cytokines from unstimulated PBMCs after 24h from NHCs, iPD patients, LRRK2-PD patients, and GBA-PD patients. (A) Secretion of IFN‐γ (Cohort p = 0.5862). (B) Secretion of IL-10 (Cohort p = 0.7260). (C) Secretion of IL-12p70 (Cohort p = 0.0377). (D) Secretion of IL-13 (Cohort p = 0.8943). (E) Secretion of IL-1β (Cohort p = 0.2654). (F) Secretion of IL-2 (Cohort p = 0.3061). (G) Secretion of IL-4 (Cohort p = 0.1340). (H) Secretion of IL-6 (Cohort p = 0.0559). (I) Secretion of IL-8 (Cohort p = 0.6352). (J) Secretion of TNF (Cohort p = 0.2755). Bars represent mean +/- SEM. NHCs, n = 35; iPD, n = 28; LRRK2-PD, n = 11; GBA-PD, n = 16. Each symbol represents the measurement from a single individual. The results in A-J were analyzed using one-way ANOVA with Tukey’s corrections for multiple comparisons. Statistically significant differences between cohorts defined by p < 0.05 are shown.
