# Supplementary material for: Parkinson's‐Linked LRRK2 and GBA1 Mutations Modulate the Peripheral Immune Response to Pseudomonas aeruginosa
Source: Mov Disord. 2025 Nov 19;41(3):651–66. doi: 10.1002/mds.70123 (PMC13022586; doi:10.1002/mds.70123)
Supplement: Supplementary file 4 — Figure S4. [file MDS-41-651-s001.pptx]

## Slide 1
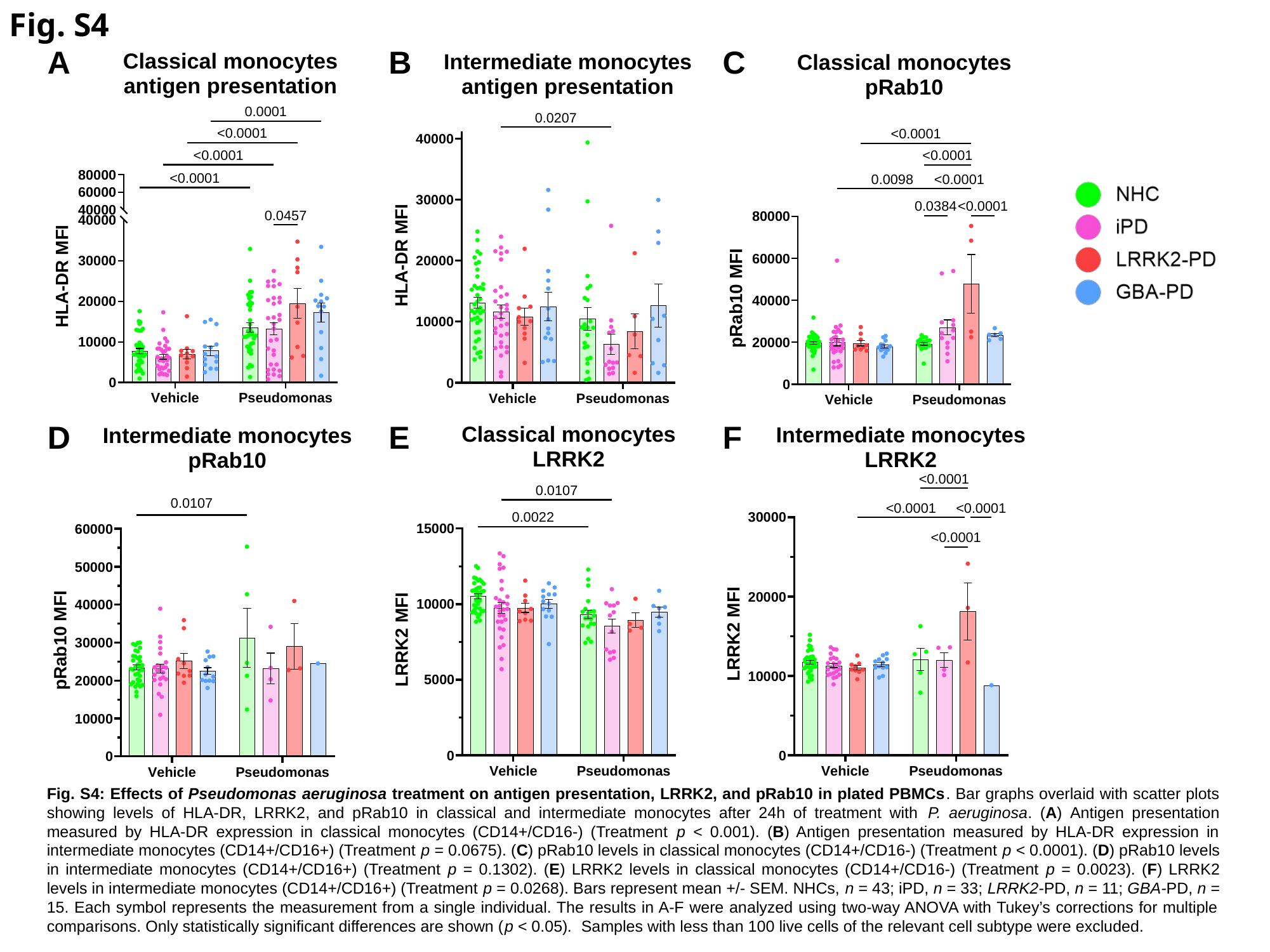

Fig. S4
A
B
C
D
E
F
Fig. S4: Effects of Pseudomonas aeruginosa treatment on antigen presentation, LRRK2, and pRab10 in plated PBMCs. Bar graphs overlaid with scatter plots showing levels of HLA-DR, LRRK2, and pRab10 in classical and intermediate monocytes after 24h of treatment with P. aeruginosa. (A) Antigen presentation measured by HLA-DR expression in classical monocytes (CD14+/CD16-) (Treatment p < 0.001). (B) Antigen presentation measured by HLA-DR expression in intermediate monocytes (CD14+/CD16+) (Treatment p = 0.0675). (C) pRab10 levels in classical monocytes (CD14+/CD16-) (Treatment p < 0.0001). (D) pRab10 levels in intermediate monocytes (CD14+/CD16+) (Treatment p = 0.1302). (E) LRRK2 levels in classical monocytes (CD14+/CD16-) (Treatment p = 0.0023). (F) LRRK2 levels in intermediate monocytes (CD14+/CD16+) (Treatment p = 0.0268). Bars represent mean +/- SEM. NHCs, n = 43; iPD, n = 33; LRRK2-PD, n = 11; GBA-PD, n = 15. Each symbol represents the measurement from a single individual. The results in A-F were analyzed using two-way ANOVA with Tukey’s corrections for multiple comparisons. Only statistically significant differences are shown (p < 0.05). Samples with less than 100 live cells of the relevant cell subtype were excluded.
