# Supplementary material for: Parkinson's‐Linked LRRK2 and GBA1 Mutations Modulate the Peripheral Immune Response to Pseudomonas aeruginosa
Source: Mov Disord. 2025 Nov 19;41(3):651–66. doi: 10.1002/mds.70123 (PMC13022586; doi:10.1002/mds.70123)
Supplement: Supplementary file 5 — Figure S5. [file MDS-41-651-s007.pptx]

## Slide 1
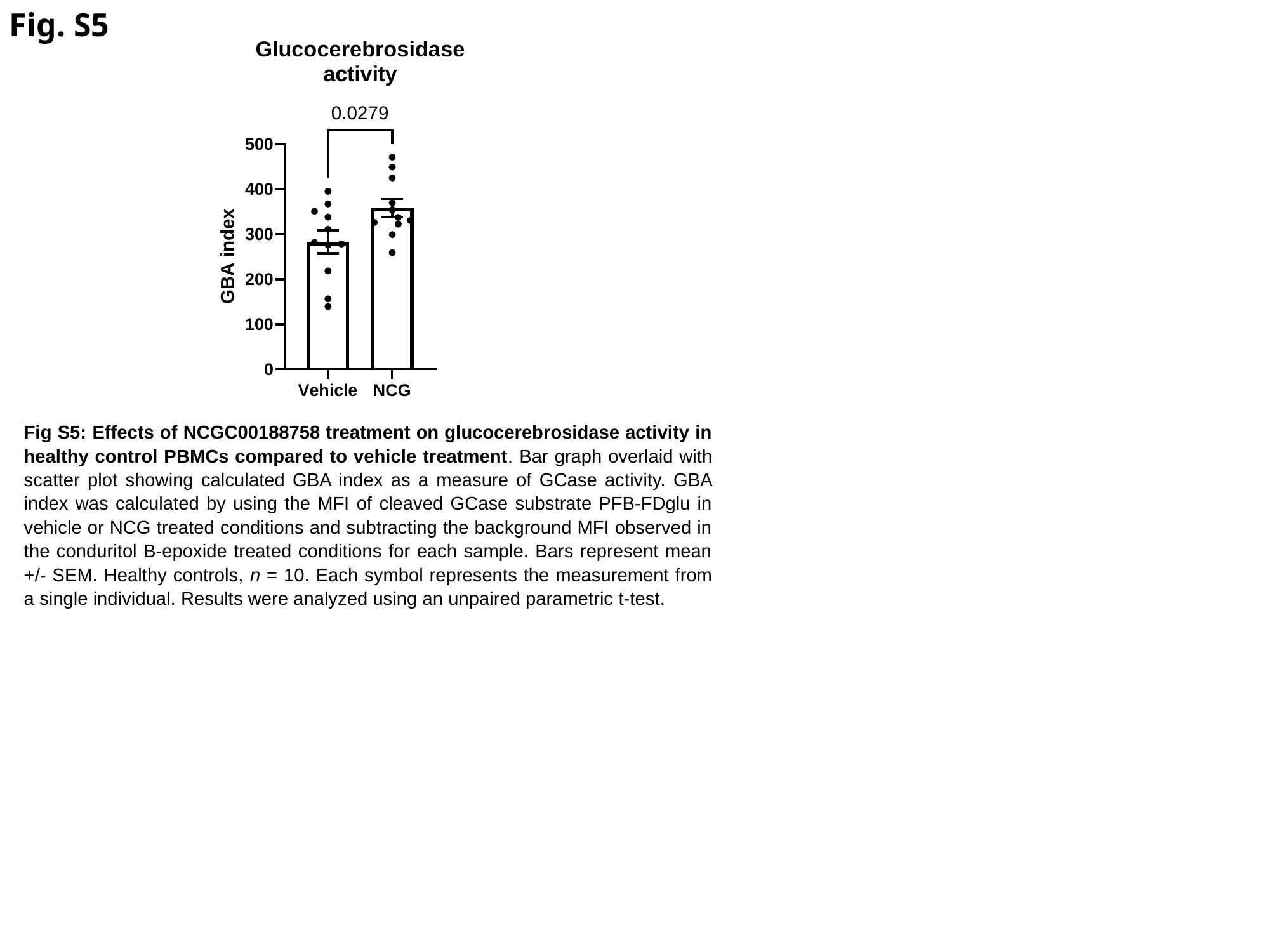

Fig. S5
Fig S5: Effects of NCGC00188758 treatment on glucocerebrosidase activity in healthy control PBMCs compared to vehicle treatment. Bar graph overlaid with scatter plot showing calculated GBA index as a measure of GCase activity. GBA index was calculated by using the MFI of cleaved GCase substrate PFB-FDglu in vehicle or NCG treated conditions and subtracting the background MFI observed in the conduritol B-epoxide treated conditions for each sample. Bars represent mean +/- SEM. Healthy controls, n = 10. Each symbol represents the measurement from a single individual. Results were analyzed using an unpaired parametric t-test.
