# Supplementary material for: Parkinson's‐Linked LRRK2 and GBA1 Mutations Modulate the Peripheral Immune Response to Pseudomonas aeruginosa
Source: Mov Disord. 2025 Nov 19;41(3):651–66. doi: 10.1002/mds.70123 (PMC13022586; doi:10.1002/mds.70123)
Supplement: Supplementary file 6 — Figure S6. [file MDS-41-651-s002.pptx]

## Slide 1
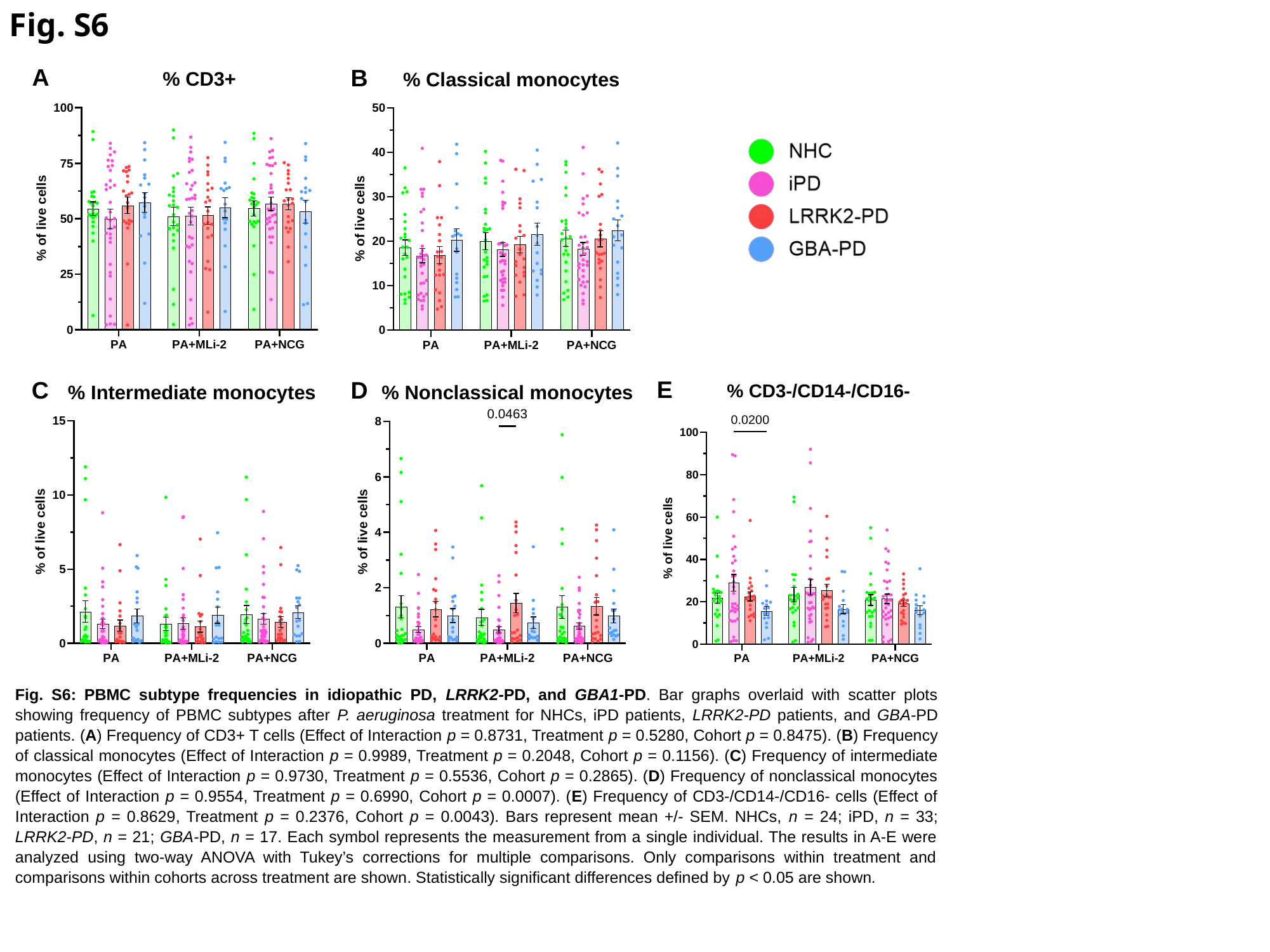

Fig. S6
A
B
E
C
D
Fig. S6: PBMC subtype frequencies in idiopathic PD, LRRK2-PD, and GBA1-PD. Bar graphs overlaid with scatter plots showing frequency of PBMC subtypes after P. aeruginosa treatment for NHCs, iPD patients, LRRK2-PD patients, and GBA-PD patients. (A) Frequency of CD3+ T cells (Effect of Interaction p = 0.8731, Treatment p = 0.5280, Cohort p = 0.8475). (B) Frequency of classical monocytes (Effect of Interaction p = 0.9989, Treatment p = 0.2048, Cohort p = 0.1156). (C) Frequency of intermediate monocytes (Effect of Interaction p = 0.9730, Treatment p = 0.5536, Cohort p = 0.2865). (D) Frequency of nonclassical monocytes (Effect of Interaction p = 0.9554, Treatment p = 0.6990, Cohort p = 0.0007). (E) Frequency of CD3-/CD14-/CD16- cells (Effect of Interaction p = 0.8629, Treatment p = 0.2376, Cohort p = 0.0043). Bars represent mean +/- SEM. NHCs, n = 24; iPD, n = 33; LRRK2-PD, n = 21; GBA-PD, n = 17. Each symbol represents the measurement from a single individual. The results in A-E were analyzed using two-way ANOVA with Tukey’s corrections for multiple comparisons. Only comparisons within treatment and comparisons within cohorts across treatment are shown. Statistically significant differences defined by p < 0.05 are shown.
