# Supplementary material for: Parkinson's‐Linked LRRK2 and GBA1 Mutations Modulate the Peripheral Immune Response to Pseudomonas aeruginosa
Source: Mov Disord. 2025 Nov 19;41(3):651–66. doi: 10.1002/mds.70123 (PMC13022586; doi:10.1002/mds.70123)
Supplement: Supplementary file 7 — Figure S7. [file MDS-41-651-s004.pptx]

## Slide 1
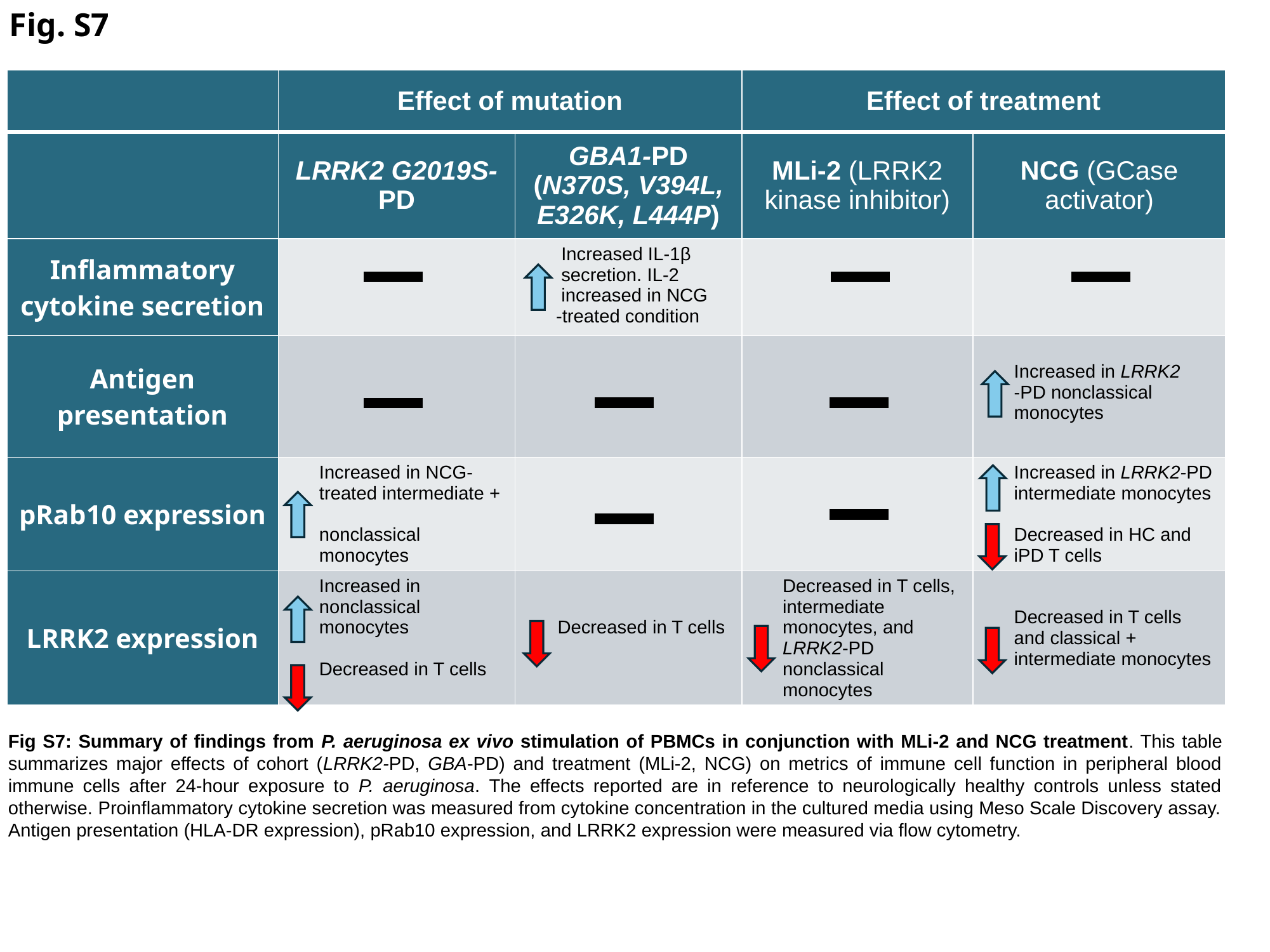

Fig. S7
| | Effect of mutation | | Effect of treatment | |
| --- | --- | --- | --- | --- |
| | LRRK2 G2019S-PD | GBA1-PD (N370S, V394L, E326K, L444P) | MLi-2 (LRRK2 kinase inhibitor) | NCG (GCase activator) |
| Inflammatory cytokine secretion | | Increased IL-1β secretion. IL-2 increased in NCG -treated condition | | |
| Antigen presentation | | | | Increased in LRRK2 -PD nonclassical monocytes |
| pRab10 expression | Increased in NCG- treated intermediate + nonclassical monocytes | | | Increased in LRRK2-PD intermediate monocytes Decreased in HC and iPD T cells |
| LRRK2 expression | Increased in nonclassical monocytes Decreased in T cells | Decreased in T cells | Decreased in T cells, intermediate monocytes, and LRRK2-PD nonclassical monocytes | Decreased in T cells and classical + intermediate monocytes |
Fig S7: Summary of findings from P. aeruginosa ex vivo stimulation of PBMCs in conjunction with MLi-2 and NCG treatment. This table summarizes major effects of cohort (LRRK2-PD, GBA-PD) and treatment (MLi-2, NCG) on metrics of immune cell function in peripheral blood immune cells after 24-hour exposure to P. aeruginosa. The effects reported are in reference to neurologically healthy controls unless stated otherwise. Proinflammatory cytokine secretion was measured from cytokine concentration in the cultured media using Meso Scale Discovery assay. Antigen presentation (HLA-DR expression), pRab10 expression, and LRRK2 expression were measured via flow cytometry.
