# Supplementary material for: Parkinson's‐Linked LRRK2 and GBA1 Mutations Modulate the Peripheral Immune Response to Pseudomonas aeruginosa
Source: Mov Disord. 2025 Nov 19;41(3):651–66. doi: 10.1002/mds.70123 (PMC13022586; doi:10.1002/mds.70123)
Supplement: Supplementary file 8 — Table S1. [file MDS-41-651-s006.pptx]

## Slide 1
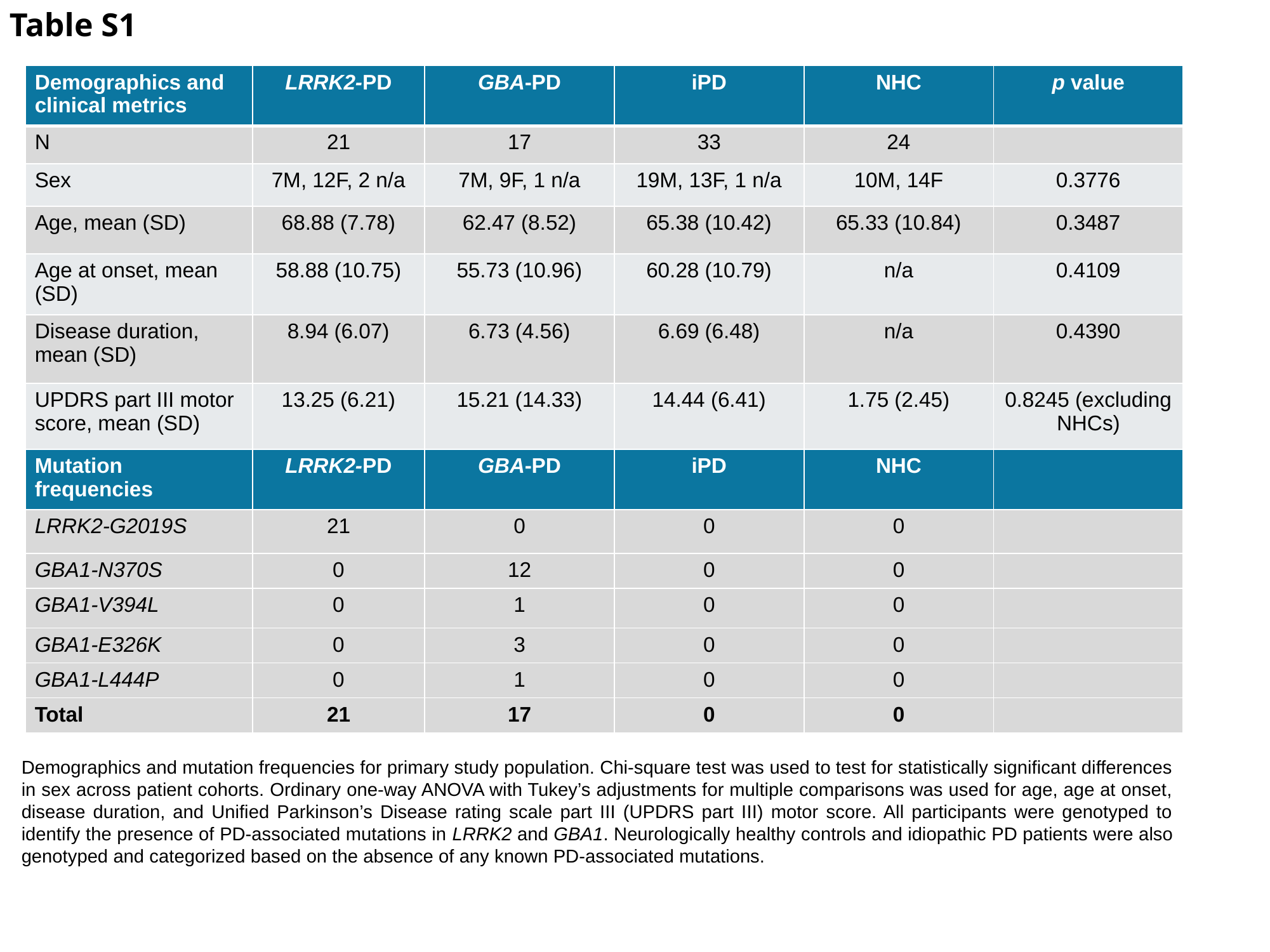

Table S1
| Demographics and clinical metrics | LRRK2-PD | GBA-PD | iPD | NHC | p value |
| --- | --- | --- | --- | --- | --- |
| N | 21 | 17 | 33 | 24 | |
| Sex | 7M, 12F, 2 n/a | 7M, 9F, 1 n/a | 19M, 13F, 1 n/a | 10M, 14F | 0.3776 |
| Age, mean (SD) | 68.88 (7.78) | 62.47 (8.52) | 65.38 (10.42) | 65.33 (10.84) | 0.3487 |
| Age at onset, mean (SD) | 58.88 (10.75) | 55.73 (10.96) | 60.28 (10.79) | n/a | 0.4109 |
| Disease duration, mean (SD) | 8.94 (6.07) | 6.73 (4.56) | 6.69 (6.48) | n/a | 0.4390 |
| UPDRS part III motor score, mean (SD) | 13.25 (6.21) | 15.21 (14.33) | 14.44 (6.41) | 1.75 (2.45) | 0.8245 (excluding NHCs) |
| Mutation frequencies | LRRK2-PD | GBA-PD | iPD | NHC | |
| LRRK2-G2019S | 21 | 0 | 0 | 0 | |
| GBA1-N370S | 0 | 12 | 0 | 0 | |
| GBA1-V394L | 0 | 1 | 0 | 0 | |
| GBA1-E326K | 0 | 3 | 0 | 0 | |
| GBA1-L444P | 0 | 1 | 0 | 0 | |
| Total | 21 | 17 | 0 | 0 | |
Demographics and mutation frequencies for primary study population. Chi-square test was used to test for statistically significant differences in sex across patient cohorts. Ordinary one-way ANOVA with Tukey’s adjustments for multiple comparisons was used for age, age at onset, disease duration, and Unified Parkinson’s Disease rating scale part III (UPDRS part III) motor score. All participants were genotyped to identify the presence of PD-associated mutations in LRRK2 and GBA1. Neurologically healthy controls and idiopathic PD patients were also genotyped and categorized based on the absence of any known PD-associated mutations.
